# Benzylisoquinoline Alkaloids from the Stems of *Limacia scandens* and Their Potential as Autophagy Inhibitors

**DOI:** 10.3390/ph15111332

**Published:** 2022-10-28

**Authors:** Hee-Ju Lee, Eun-Jin Park, Byeol Ryu, Hyo-Moon Cho, Duc-Trong Nghiem, Ha-Thanh-Tung Pham, Cheol-Ho Pan, Won-Keun Oh

**Affiliations:** 1 Natural Product Informatics Research Center, Korea Institute of Science and Technology, Gangneung 25451, Korea; 2 Korea Bioactive Natural Material Bank, Research Institute of Pharmaceutical Sciences, College of Pharmacy, Seoul National University, Seoul 08826, Korea; 3 Department of Botany, Hanoi University of Pharmacy, Hanoi 000084, Vietnam; 4 Faculty of Pharmacy, PHENIKAA University, Yen Nghia, Ha Dong, Hanoi 12116, Vietnam

**Keywords:** *Limacia scandens*, dimeric benzylisoquinoline alkaloids, autophagy, inhibitor

## Abstract

*Limacia scandens* is traditionally used to treat depression and affective disorders in Malaysia. The chemical compositions have been reported to include bisbenzylisoquinoline and aporphine-type alkaloids in the genus *Limacia* Lour., but studies on the components of *L. scandens* have rarely been reported. Therefore, this study was conducted to determine new benzylisoquinoline alkaloid derivatives with autophagy regulation activity from this plant. Bioactivity-guided isolation was applied to various column chromatography methods using RP-18, Sephadex LH-20 open column chromatography, and preparative HPLC. The chemical structures of the isolated compounds were elucidated through spectroscopic data analysis, including NMR, HR-ESI-MS, and ECD data. In addition, isolated compounds were tested for autophagy-regulating activity in HEK293 cells expressing GFP-L3. Three new dimeric benzylisoquinoline alkaloids (**1**−**3**), one new 4-hydroxybenzoic acid-conjugated benzylisoquinoline alkaloid (**4**), and six known compounds (**5**−**10**) were isolated from the stems of *L. scandens*. All compounds (**1**–**10**) were screened for autophagy regulation in HEK293 cells stably expressing the GFP-LC3 plasmid. Among the isolated compounds, **1**, **2**, and **4** showed autophagic regulation activity that blocked the process of combining autophagosomes and lysosomes. They also inhibit the protein degradation process from the autolysosome as inhibitors of autophagy. Novel benzylisoquinoline alkaloids from *L. scandens* showed potent potency for the inhibition of autophagic flux. This study provides potential candidates for developing natural autophagy inhibitors for disease prevention and treatment.

## 1. Introduction

Autophagy is one of the essential mechanisms for maintaining the homeostasis of various organs through the degradation of dysfunctional organelles, proteins, and intracellular pathogens for their removal or utilization [1]. The formation of an autophagosome, a double-membrane vesicle that forms in mammalian cells, is required for autophagy to function. Formed autophagosomes merge with lysosomes to destroy the cellular contents [2]. Autophagy serves as a net scavenger for protein fragments and poisons that may harm human health. However, the malfunction or overactivation of autophagy can result in various diseases, including cancer, infection, and neurodegenerative disorders (Parkinson’s and Alzheimer’s disease) [3]. The hyperactivation of autophagy also causes muscle loss and frailty in muscular-related diseases [4]. Therefore, several studies have revealed that autophagy regulators can aid in treating human autophagy-related disorders. Recent studies have shown that resveratrol, a polyphenolic stilbene in grapes, stimulates AMPK and SIRT1 to increase autophagy [5]. Curcumin decreases neurotoxicity by promoting α-synuclein degradation mediated by autophagy [6]. 6-Gingerol, which has an anticancer effect, enhanced tumor necrosis factor-related apoptosis-inducing ligand (TRAIL)-induced apoptosis by blocking autophagic flux [7]. Thus, phytochemicals derived from natural products as autophagy activators or inhibitors may be a promising strategy for preventing and treating various diseases. 

*Limacia scandens* Lour. belongs to the family Menispermaceae and is distributed sporadically in lowland forests and swamps in many regions of Southeast Asia, including southern Myanmar, Indonesia, Thailand, the western Malaysian peninsula, and Vietnam [8]. The plant grows well in open habitats and secondary forests from low altitudes up to 150 m and woody climbers of 1–2 m in length. The leaves are alternate, stipules are absent, and the petiole is swollen at the base and at the apex. The leaf blade is simple and has a pinnate shape. Inflorescences have 2–6 densely flowered cymes, and the flowers are unisexual, usually small, inconspicuous, and mostly pedicellate. In addition, the ripe fruit has a sour taste and can be edible. The most popular species, *L. oblonga* (Mier.), has been used to treat sore eyes and hemafecia as a traditional herbal medicine in Malaysia [9]. Bisbenzylisoquinoline, proaporphine, oxoaporphine, and hasubanan-type alkaloids have been reported in the genus Limacia [10,11]. However, even though the stems and roots of *L. scandens* are known to have therapeutic intestinal disease activity and sympathomimetic activity [12], there are few reports of the chemical composition and biological activity of *L. scandens*.

In this study, we investigated a 70% ethanol extract of the stems of *L. scandens* and isolated ten compounds (**1**−**10**), including three new dimeric benzylisoquinoline alkaloids (**1**−**3**), one new 4-hydroxybenzoic acid conjugated benzylisoquinoline alkaloid (**4**), and six known compounds (**5**−**10**). Among isolates, **1**, **2**, and **4** showed autophagic regulation activity that blocked the process of combining autophagosomes and lysosomes. In addition, these compounds inhibited the protein degradation process from the autolysosome as an autophagy inhibitor in HEK293 cells expressing GFP-L3. 

## 2. Results

### 2.1. Structure Determination of ***1***−***4*** from L. scandens

**Figure 1:**
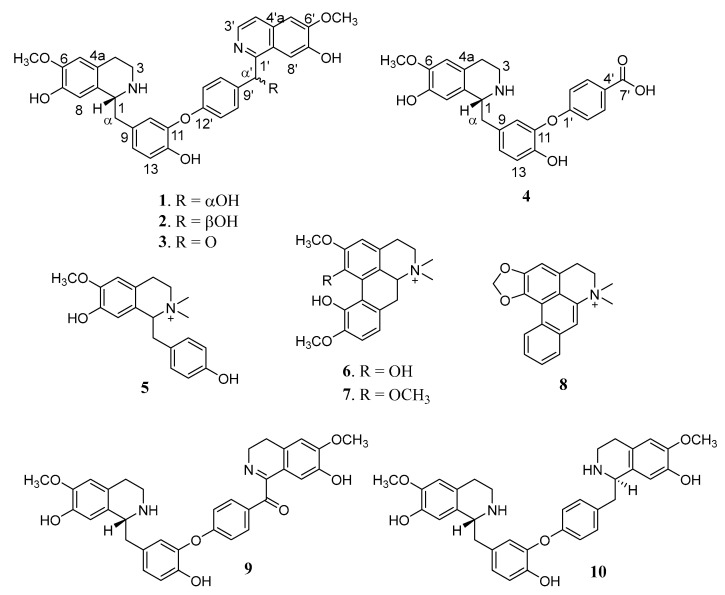
Chemical structures of isolated compounds **1**–**10** from *L. scandens*.

The 70% EtOH extract of the dried stems of *L. scandens* was subjected to RP-18, Sephadex LH-20 open column chromatography and semipreparative HPLC to obtain three new bisbenzylisoquinoline-type alkaloids (**1**–**3**) and one new benzylisoquinoline-type alkaloid (**4**), together with six known compounds (Figure 1). 

#### 2.1.1. Limaciascadine A (**1**)

**Figure 2:**
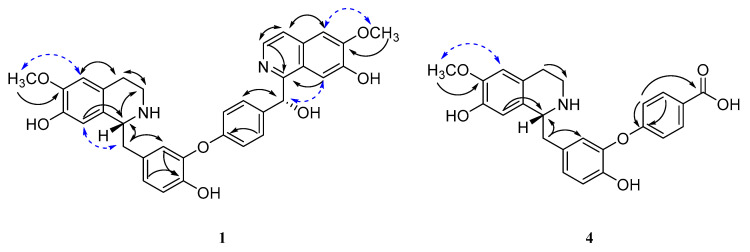
The key HMBC correlations (black) and ROESY correlations (blue) of **1**–**4**.

**Figure 3:**
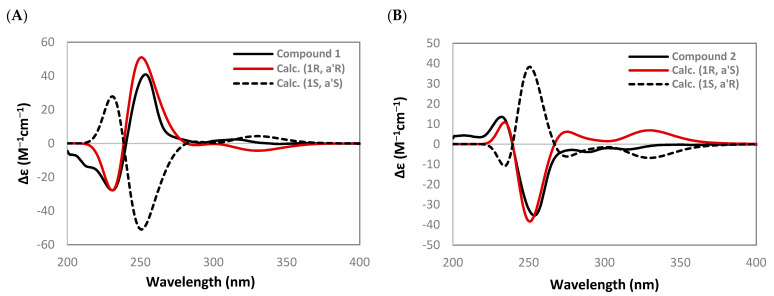
Experimental and calculated CD spectra of **1** (**A**) and **2** (**B**).

Compound **1**, limaciascadine A, was obtained as a brownish gum with ${\left[\alpha \right]}_{D}^{20}$ +23 (*c* 0.2, MeOH). The molecular formula of **1** was proposed as C_34_H_32_N_2_O_7_ based on a pronated molecular ion at *m/z* 581.2280 [M + H]^+^ (calcd for C_34_H_33_N_2_O_7_, 581.2288) in the HRESIMS. The ^1^H NMR data of **1** showed one A_2_B_2_ aromatic proton signal at *δ*h 7.33 (2H, d, *J* = 8.4 Hz, H-10′, 14′) and 6.91 (2H, d, *J* = 8.4 Hz, H-11′, 13′), one ABX aromatic proton signal at *δ*h 6.78 (1H, s, H-10), 6.96 (1H, d, *J* = 8.2 Hz, H-13) and 7.03 (1H, d, *J* = 7.4 Hz, H-14), two *para* aromatic proton signals at *δ*h 6.47 (1H, s, H-8), 6.69 (1H, s, H-5), 7.52 (1H, s, H-8′) and 7.63 (1H, s, H-5′), AB quartet aromatic proton signals at *δ*h 8.15 (1H, d, *J* = 6.0 Hz, H-4′) and 8.27 (1H, d, *J* = 6.3 Hz, H-3′), and two methoxy groups at *δ*h 3.79 (3H, s) and 4.11 (3H, s). The ^13^C NMR data of **1** indicated 34 carbon signals, which included one oxygenated methine carbon at *δ*c 71.5 (C-α′), AB quartet aromatic carbons at *δ*c 128.9 (C-3′) and 124.1 (C-4′), one imine carbon at *δ*c 156.4 (C-1′), and two aromatic *O*-methyl groups at *δ*c 56.4 (6-OCH_3_) and 57.4 (6′-OCH_3_) (Table 1). The structure of the A moiety was assigned from the signals of H-10 and H-14 to C-11 (*δ*c 144.3) and C-12 (*δ*c 150.1); from *para* aromatic protons of H-5 to C-4 (*δ*c 25.8), C-6 (*δ*c 149.3), C-7 (*δ*c 146.6) and C-8a (*δ*c 124.7); from H-8 to C-α (*δ*c 40.2), C-4a (*δ*c 123.6), C-6 and C-7; the aliphatic protons of H-4 (*δ*h 2.98) to C-3 (*δ*c 40.4), C-5 (*δ*c 112.6) and C-8a; from H-3 (*δ*h 3.45) to C-1 (*δ*c 57.4) and C-4; and from H-α (*δ*h 2.98 and 3.26) to C-1 and C-10 (*δ*c 124.1). The structure of the B group was elucidated by the HMBC from the aromatic protons signals of H-10′ to C-α′ and C-12′ (*δ*c 160.1); of H-5′ to C-4′, C-6′ (*δ*c 158.3) and C-7′ (*δ*c 152.4); of H-8′ to C-1′ (*δ*c 156.4) and C-4′a (*δ*c 137.9); the AB quartet aromatic protons of H-3′ to C-1′, C-4′ and C-4′a; of H-4′ to C-3′, C-5′ (*δ*c 107.5) and C-8′a (*δ*c 122.1); the oxygenated methine proton of H-α′ (*δ*h 6.60) to C-1′, C-9′ (*δ*c 135.3) and C-10′ (*δ*c 130.2); and the *O*-methyl protons at *δ*h 3.79 and 4.11 (3H, s) to C-6 and C-6′, respectively. The ROESY spectrum showed correlations of H-5/6-OCH_3_, H-8/H-α, H-5′/6′-OCH_3_, and H-8′/H-α′ (Figure 2). Compound **1** was a C-α hydroxy bisbenzylisoquinoline alkaloid, supported by their deshielded chemical shifts at a linkage between C-11 and C-12′ of the two benzylisoquinoline moieties [13]. The absolute configuration of **1** was calculated using TDDFT at the 6-31G/B3LYP level according to the Boltzmann distribution. The experimental ECD spectrum of 1*R*,α′*R* closely matches the calculated ECD spectrum (Figure 3A). Therefore, the structure of limaciascadine A was assigned as shown. 

#### 2.1.2. Limaciascadine B (**2**)

Compound **2**, limaciascadine B, was obtained as a brownish gum with ${\left[\alpha \right]}_{D}^{20}$ −37 (*c* 0.2, MeOH). The molecular formula of **2** was deduced as C_34_H_32_N_2_O_7_ at m/z 581.2265 [M + H]^+^ (calculated for C_34_H_33_N_2_O_7_, 581.2288) from a positive ion peak of HRESIMS. The ^1^H and ^13^C NMR data were similar to those of **1**, suggesting that **2** is an isomer of **1**. The absolute configuration of **2** was determined as 1*R*,α′*S* by comparing the experimental ECD spectrum that calculated the ECD spectrum for 1*R*,α′*S* and 1*S*,α′*R* (Figure 3B). 

#### 2.1.3. Limaciascadine C (**3**)

Compound **3**, limaciascadine C, was isolated as a brownish gum with ${\left[\alpha \right]}_{D}^{20}$ −9 (*c* 0.2, MeOH). Its molecular formula, C_34_H_30_N_2_O_7_, was shown on the basis of a pronated molecular ion at *m/z* 579.2120 [M + H]^+^ (calcd for C_34_H_31_N_2_O_7_, 579.2131) in the HRESIMS, which was 2 amu less than that of **1**. The ^1^H and ^13^C NMR data (Table 1) were similar to those of **1**, replacing the carbonyl carbon at the α′ position. The carbonyl group at C-α′ (*δ*c 193.5) was confirmed based on HMBC correlations from H-10′ and 14′ (*δ*h 7.77, each 1H, d, *J* = 8.8 Hz) to C-α′, suggesting that **3** is a 3′,4′-dehydro-linderegatine. The absolute configuration of **3** was determined as 1*R*, which compared the experimental CD spectrum to the calculated ECD spectra (Figure S27). Limasiascadine C was defined as (1*R*)-3′,4′-dehydro-linderegatine. 

#### 2.1.4. Limaciascadine D (**4**)

Compound **4**, limaciascadine D, was obtained as a brownish gum with ${\left[\alpha \right]}_{D}^{20}$ −8 (*c* 0.2, MeOH). The molecular formula of **4** was suggested as C_24_H_23_NO_6_, which was [M + H]^+^ ion at *m/z* 422.1611 (calcd for C_24_H_24_NO_6_, 422.1604) in the HRESIMS. The ^1^H NMR spectrum of **4** showed one A_2_B_2_ aromatic proton signal at *δ*h 7.87 (2H, br s, H-2′, 6′) and 6.88 (2H, br s, H-3′, 5′), one ABX aromatic proton signal at *δ*h 6.93 (1H, overlapped, H-10), 6.94 (1H, overlapped, H-13) and 7.00 (1H, d, *J* = 7.9 Hz, H-14), two *para* aromatic protons at *δ*h 6.55 (1H, s, H-8) and 6.59 (1H, s, H-5) and one methoxy group at *δ*h 3.71 (3H, s). The ^13^C NMR data of **4** indicated 24 carbon signals composing one carboxylic carbon at *δ*c 167.4 (C-7′) and one methoxy group at *δ*c 55.5 (6-OMe). ^1^H and ^13^C NMR data suggested that **4** comprised one benzylisoquinoline alkaloid and one 4-hydroxybenzoic acid in the structure. The structure of **4** was elucidated by the HMBC correlations from proton signals of H-8 to C-6 (*δ*c 146.2), C-7 (*δ*c 144.3), C-4a (*δ*c 124.7) and C-1 (*δ*c 55.7); of H-2′, 6′ to C-7′, C-1′ (*δ*c 161.4) and C-3′, 5′ (*δ*c 115.3); of H-3′, 5′ to C-1′ and C-4′ (*δ*c 125.3); of H-α (*δ*h 2.78 and 2.97) to C-1 and C-10 (*δ*c 123.5); and of one methoxyl group at *δ*h 3.71 (3H, s) to C-6. The methoxyl group was located at C-5 from ROESY correlation data between H-5 and 6-OCH_3_ (Figure 2). The linkage between C-11 of benzylisoquinoline and C-1′ of 4-hydroxybenzoic acid was confirmed by their ^13^C NMR chemical shift comparison with the literature [14]. The absolute configuration of **4** was determined to be 1*R* in the same manner as in **3** (Figure S27). Limaciascadine D was determined as shown in Figure 1. 

Additionally, six known compounds were identified as (−)-magnocurarine (**5**), (+)-magnoflorine (**6**), (+)-menisperine (**7**), 7,7-dimethyl-6,7-dihydrodioxolobenzoquinolin (**8**), (1*R*)-11-hydroxy-linderegatine (**9**), and (+)-lindoldhamine (**10**) based on their spectroscopic data with reported literature [13,14,15,16,17,18,19]. 

### 2.2. Screening of Autophagy Regulation in HEK293 Cells Stably Expressing GFP-LC3

**Figure 4:**
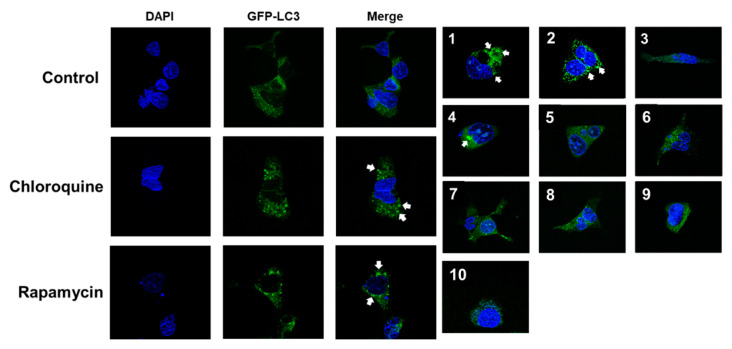
Screening of isolated alkaloids from *L. scandens* for regulation of autophagy. Compound treatment induced autophagosome formation in HEK293 cells. HEK293 cells stably expressing GFP-LC3 were incubated with 20 μM compounds for 24 h, and the formation of LC3-GFP puncta was analyzed with confocal microscopy.

Many human diseases, including cancer and neurodegenerative diseases, are linked to autophagy. To identify potential autophagy-regulating compounds, HEK293 cells stably expressing GFP-LC3 were used to screen for autophagy regulation. In HEK293 cells, chloroquine and rapamycin, which are known to inhibit and induce autophagy drugs, were used as positive controls, showing the formation of punctuates [20,21]. In the confocal microscopic image, the tested chloroquine and rapamycin exhibited the formation of puncta, and a strong GFP signal was detected in the cell cytosol. These results indicated that the formation of puncta in HEK293 cells stably expressing GFP-LC3 reveals the autophagy regulation of the compounds. Cells were treated with the isolated compounds (**1**−**10**) at 20 μM concentrations for 24 h, and the cytosol was observed under a confocal microscope (Figure 4). Compound **1** showed the strongest formation of LC3 puncta among the treated compounds, and this result was higher than that in the control group. Compound **2**, which is only different at α-OH or β-OH compared to that of **1**, showed autophagic regulation activity similar to that of **1**. Interestingly, **3** with a ketone functionality instead of α-OH or β-OH in **1** and **2** disappeared the formation of the punctuates at the same concentration. Similarly, **4** with a carboxyl group also showed strong punctuates. Thus, **1**, **2** and **4** were selected for further investigation into whether the compounds were autophagy inhibitors or inducers.

### 2.3. Upregulating the Protein Expression Levels of LC3B and p62 in HEK293 Cells

**Figure 5:**
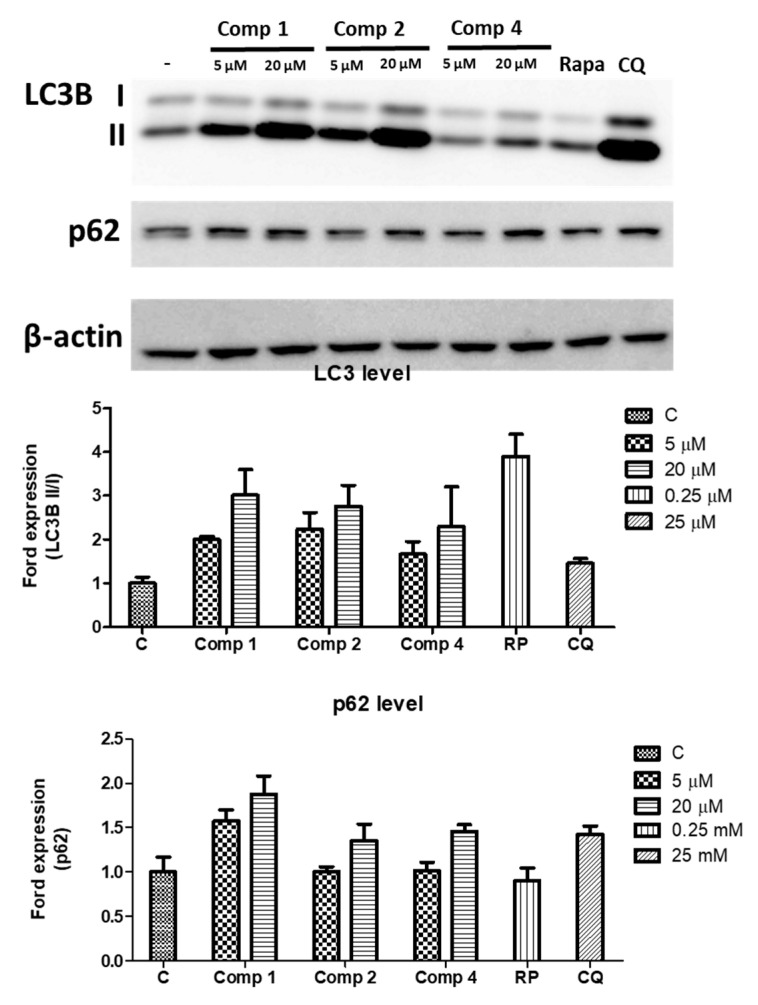
The autophagy-related protein expression level of compound-treated cells. LC3B was calculated by the LC3B II/I ratio, and the ratio was normalized to the control group (nontreated group). The p62 protein level was detected, and the expression level was divided by the control group’s protein level for normalization. (Rapa: rapamycin treatment group; CQ: chloroquine treatment group).

The autophagic system is involved in both the bulk degradation of cytosolic proteins and the selective degradation of cytoplasmic organelles. Autophagosomes fuse the engulfed substrates with the lysosomes, and the formed autolysosomes, which are active at an acidic pH, degrade the cytoplasm-derived compartments, including the inner membrane of autophagosomes [22]. During autophagy, a cytosolic form of LC3 (LC3-I) is changed to LC3-phosphatidylethanolamine conjugate (LC3-II) on the autophagosomal membranes. p62 is a ubiquitin-binding protein that is recruited into autophagosomes due to its direct interaction with LC3-II. As autophagy progresses, p62 is degraded, but when autophagy is inhibited, p62 is not degraded and appears to increase [23]. To determine whether the isolated compounds based on autophagy marker proteins induce or inhibit autophagy, changes in p62, LC3B-I, and LC3B-II as marker proteins were detected by Western blot. LC3B levels were calculated as LC3B II/I levels, which indicate the level of transition from LC3B I to II. Protein levels of p62 were also detected to monitor the degradation tendency of autophagosomes. Chloroquine and rapamycin were treated as controls for autophagy of the inhibitor and activator, respectively. The expression levels of LC3B and p62 increased in the chloroquine-treated group, an autophagy inhibitor, and these results indicated that p62, LC3B II and I were not degraded but accumulated in the cells. In the rapamycin-treated group, Western blot results showed that the levels of LC3B-I and p62 were decreased, whereas the concentration of LC3B-II was increased. Interestingly, LC3B II/I levels were increased, indicating that conversion from LC3B I to II was actively processed as autophagy was activated [24]. When the isolated **1**, **2**, and **4** were treated at concentrations of 5 μM and 20 μM, the results, which are similar to those of the chloroquine-treated group, are shown. Compound **1** showed the highest intensity at the p62 protein level, and the LC3 II level also increased strongly compared to the control group in a dose-dependent manner. Compounds **2** and **4** also showed an increase in activity similar to that of **1**, but the activity of **4** was weaker than that of **1** and **2** (Figure 5). These results strongly suggested that **1**, **2**, and **4** blocked the process of combining autophagosomes and lysosomes and inhibited the protein degradation process by the autolysosomes.

### 2.4. Blocking Autophagic Flux in HEK293 Cells Transfected with GFP-mRFP-LC3

**Figure 6:**
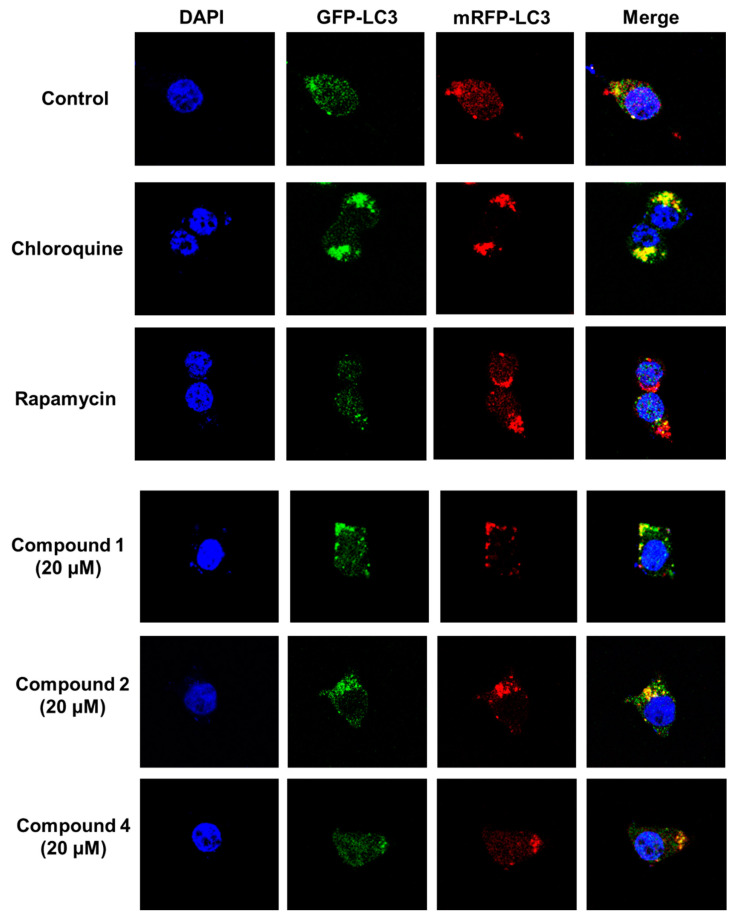
The GFP-mRFP-tagged LC3 punctuates images with the treatment of compounds. HEK293 cells were treated with autophagy-regulating **1**, **2**, and **4**. The LC3 puncta were detected using confocal microscopy.

To determine whether isolated **1**, **2**, and **4** inhibited autophagy at the cellular level, HEK293 cells were transfected with GFP-mRFP-LC3, and autophagosomes were directly analyzed by confocal microscopy. When autophagosomes are generated, both GFP and mRFP are activated, allowing the identification of yellow puncta. Rapamycin, an autophagy activator, increased the formation of autolysosomes, and the green signal of GFP was lost due to the acidic environment of the autolysosomes. As a result, the red mRFP signal strongly appeared, and these results suggested a relationship between autophagosomes and autolysosomes induced by the compounds [25]. GFP-mRFP-LC3-transfected cells were treated with the compounds at a concentration of 20 μM, and nuclear counterstaining was detected with DAPI staining. The formation of autophagosomes by the autophagy inhibitor chloroquine was detected with strong green fluorescence of GFP-LC3 and red fluorescence of mRFP-LC3 in the cytoplasm. The chloroquine-treated group showed more yellow puncta than the control group in the merged image; thus, the result demonstrated that autophagosomes were blocked by chloroquine treatment and stacked in the cytoplasm as yellow fluorescence. In contrast, the rapamycin-treated group destroyed the green fluorescence of GFP-LC3 in autolysosomes, and strong mRFP puncta were detected, which means that rapamycin treatment exerted autophagic flux. The increase in yellow puncta after treatment with isolated **1**, **2**, and **4** clearly showed an inhibitory effect on autophagic flux (Figure 6). 

## 3. Discussion

**Figure 7:**
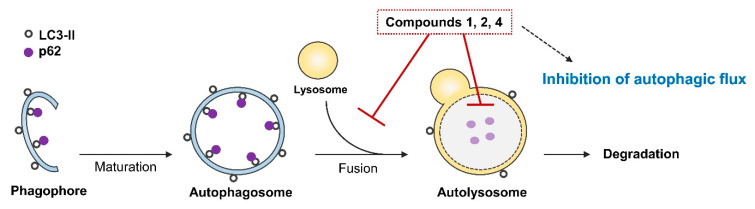
Illustration of the inhibition of autophagic flux by isolated compounds. Compounds **1**, **2**, and **4** exhibited the inhibition of autophagic flux, blocking the fusion of autophagosomes with lysosomes or inhibiting the autolysosomal protein-degradation process.

In this study, stems and roots of *L. scandens* are used in traditional medicine to have therapeutic intestinal disease activity and sympathomimetic activity [12], but there are few reports of the chemical composition and biological activity of *L. scandens*. The four novel benzylisoquinoline alkaloids along with six known compounds were isolated by various column chromatography methods from the stems of *L. scandens*. Three new dimeric benzylisoquinoline alkaloids (**1**−**3**) and one new 4-hydroxybenzoic acid conjugated benzylisoquinoline alkaloid (**4**) are novel substances isolated from this plant. Compounds **1**, **2**, and **4** had autophagic regulatory activity that prevented the process of autophagosomes and lysosomes. Additionally, these compounds acted as autophagy inhibitors in HEK293 cells expressing GFP-L3, preventing the autolysosome from degrading proteins (Figure 7). Compounds **1**−**3** are derivatives of similar structures having different structures only in the αʹ position. Compounds **1** and **2**, which differ only in α-OH and β-OH at αʹ position, showed similar activity, but compound **3**, having a ketone functionality at the same position, did not. These results revealed that compounds with similar derivatives exhibited different biological activities depending on the functional group.

The regulation of autophagy is an essential mechanism in research on disease treatment. It has been reported that autophagy inhibitors enhance the effectiveness of cancer treatment. The efficacy of TRAIL was boosted by blocking the autophagic flux after treatment with 6-gingerol. Cotreatment of ovarian cancer cells with wortmannin, a well-known autophagy inhibitor, and cisplatin increased the apoptosis-inducing effect of anticancer medications and decreased resistance to chemotherapy [26]. The aging muscles in patients with muscle atrophy or sarcopenia significantly enhanced autophagic flux and increased protein degradation in the muscles [27]. Docosahexaenoic acid (DHA) has been suggested as a treatment for sarcopenia, but few related studies have been reported. There is currently no drug development in which the exact target mechanism for sarcopenia has been identified. In this regard, the inhibitory action of autophagy could be an important drug target. Tetrandrine, a bisbenzylisoquinoline alkaloid isolated from *Stephania tetrandra*, inhibited autophagy by deacidification of lysosomes in the late stage of bladder, prostate, cervical, and pancreatic cancers [28]. Additionally, neferine isolated from *Nelumbo nucifera* promoted apoptosis through the accumulation of p62/SQSTM1 by inhibiting autophagic flux in head and neck squamous cell carcinoma [29]. We isolated and reported different types of bisbenzylisoquinoline alkaloids in this manuscript and confirmed their autophagy inhibitory activity. This result suggests the possibility of a new skeleton of a treatment candidate for anticancer drugs or sarcopenia-related drugs. 

## 4. Materials and Methods

### 4.1. General Experimental Procedures

Optical rotations were recorded on a JASCO P-2000 polarimeter (JASCO International Co., Ltd., Tokyo, Japan). IR data were collected using a Nicolet 6700 FT-IR spectrometer (Thermo Electron Corp., Waltham, MA, USA). ECD spectra were obtained using Chirascan Plus (Applied Photophysics Ltd., Surrey, United Kingdom). A Waters Xevo G2 QTOF MS spectrometer (Waters Co., Milford, MA, USA) was used for high-resolution electrospray ionization mass spectrometry (HRESIMS) values. Semipreparative HPLC experiments were performed using a Gilson HPLC system with a 321 pump and a UV/VIS-155 detector. A Phenomenex Phenyl-Hexyl column (10 × 250 mm, 5 μm particle size, USA) was used as the HPLC column. The NMR spectra for 1D (^1^H and ^13^C) and 2D (HSQC, HMBC, NOESY, and ROESY) were measured on a Varian Unity Inova spectrometer at 500 MHz (Agilent Technologies, Santa Clara, CA) and an AVANCE spectrometer at 800 MHz (Bruker, Germany). YMC*Gel ODS-A and Sephadex LH-20 were used for column chromatography. Thin-layer chromatography experiments were performed with silica gel 60 F_254_ and RP-18 F_254_ plates. A Gilson semi-Prep HPLC system was composed of a flow rate of 3 mL/min and UV detection at 201 and 254 nm. All solvents (Dae Jung Pure Chemical, Siheung, Korea) for extraction and isolation were of analytical grade.

### 4.2. Plant Material 

The dried stems of the plant were collected in the Ba Vi district (21°05′04.1″ N 105°20′00.3″ E) in 2017. Based on morphological characteristics, Duc Trong Nghiem of the Department of Botany, Hanoi University of Pharmacy, Hanoi, Vietnam, identified the samples as *Limacia scandens* Lour (Figure S29). Accordingly, a voucher specimen was deposited with the accession number HNIP.18510/16 at the Medicinal Herbarium of Hanoi University of Pharmacy (HNIP). 

### 4.3. Extraction and Isolation 

Dried stems of *L. scandens* (3 kg) were extracted with 70% EtOH for 4 h by ultrasonication, and the combined extracts after filtration were evaporated in a vacuum. The 70% EtOH extract (280 g) was suspended in distilled water and partitioned with *n*-hexane, EtOAc, and *n*-BuOH. The BuOH fraction (50 g) was separated on an RP-18 column and eluted with aqueous MeOH to give 26 fractions (fractions 1–26). Fraction 8 (2 g) was separated by Sephadex LH-20 (3.5 × 65 cm, MeOH), and fraction 8.2 (100 mg) was isolated by HPLC [mobile phase MeCN in H_2_O containing 0.1% formic acid (0–27 min, isocratic 18% MeCN; 28–50 min, 18–30% MeCN)] to yield **1** (t_R_ = 25 min) and **2** (t_R_ = 28 min). Fractions 9 and 10 (900 mg) were rechromatographed using Sephadex LH-20 (3.5 × 65 cm, 70% MeOH) column chromatography to obtain seven subfractions (9.1–9.7). Compounds **3**, **9** and **10** from fraction 9.6 were reisolated by HPLC [mobile phase MeCN in H_2_O containing 0.1% formic acid (0–5 min, 15% MeCN; 5–40 min, 15–25% MeCN)] to yield **3** (t_R_ = 20 min), **9** (t_R_ = 25 min) and **10** (t_R_ = 35 min). Fraction 12 (1 g) using a Sephadex LH-20 column was eluted with 70% MeOH to give five fractions (12.1–12.5). Fraction 12.4 was purified by HPLC [mobile phase MeCN in H_2_O containing 0.1% formic acid (0–5 min, 20% MeCN; 5–50 min, 20–25% MeCN)] to obtain **5** (t_R_ = 35 min) and **8** (t_R_ = 45 min). Fraction 15 (2 g) was subjected to open column chromatography using a Sephadex LH-20 column (3.5 × 65 cm, MeOH) to obtain subfractions 15.1–15.6. Fraction 15.3 was isolated using HPLC [mobile phase MeCN in H_2_O containing 0.1% formic acid (0–50 min, isocratic 23% MeCN)] to obtain **6** (t_R_ = 35 min) and **7** (t_R_ = 57 min). Fraction 15.4 was purified by HPLC [mobile phase MeCN in H_2_O containing 0.1% formic acid (0–40 min, isocratic 25% MeCN)] to obtain **4** (t_R_ = 35 min). 

### 4.4. Spectroscopic and Physical Characteristic of Compounds

*Limaciascadine A (**1**)*: Brownish gum; ${\left[\alpha \right]}_{D}^{20}$ +23 (*c* 0.2, MeOH); UV (MeOH) λ_max_ (log *ε*) 203 (3.3), 229 (3.0), 253 (3.1), 285 (2.5), 315 (2.3) nm; IR *ν*_max_ 1672, 1508, 1433, 1278, 1200, 1137 cm^−1^; ECD (MeOH) λ_max_ (Δ*ε*) 232 (−27.4), 255 (40.1), 292 (0.3), 315 (2.4) nm; ^1^H and ^13^C NMR, see Table 1; HRESIMS *m/z* 581.2280 [M + H]^+^ (calcd for C_34_H_33_N_2_O_7_, 581.2288). 

*Limaciascadine B (**2**)*: Brownish gum; ${\left[\alpha \right]}_{D}^{20}$ −37 (*c* 0.2, MeOH); UV (MeOH) λ_max_ (log *ε*) 204 (3.3), 230 (3.1), 255 (3.1), 283 (2.6), 315 (2.3) nm; IR *ν*_max_ 1671, 1508, 1432, 1278, 1199, 1136 cm^−1^; ECD (MeOH) λ_max_ (Δ*ε*) 233 (13.4), 254 (−35.4), 290 (−4.0), 302 (−2.0) nm; ^1^H and ^13^C NMR, see Table 1; HRESIMS *m/z* 581.2265 [M + H]^+^ (calcd for C_34_H_33_N_2_O_7_, 581.2288).

*Limaciascadine C (**3**)*: Brownish gum; ${\left[\alpha \right]}_{D}^{20}$ −9 (*c* 0.2, MeOH); UV (MeOH) λ_max_ (log *ε*) 202 (3.3), 235 (3.2), 290 (2.8) nm; IR *ν*_max_ 1671, 1515, 1433, 1285, 1200, 1159, 1137.8 cm^−1^; ECD (MeOH) λ_max_ (Δ*ε*) 231 (−6.6), 286 (−1.4), 302 (0.4) nm; ^1^H and ^13^C NMR, see Table 1; HRESIMS *m/z* 579.2120 [M + H]^+^ (calcd for C_34_H_31_N_2_O_7_, 579.2131).

*Limaciascadine D (**4**)*: Brownish gum; ${\left[\alpha \right]}_{D}^{20}$ −8 (*c* 0.2, MeOH); UV (MeOH) λ_max_ (log *ε*) 202 (3.2), 230 (2.7), 250 (2.5), 282 (2.4) nm; IR *ν*_max_ 1678, 1516, 1433, 1272, 1202, 1139 cm^−1^; ECD (MeOH) λ_max_ (Δ*ε*) 229 (−5.6), 278 (0.1), 291 (−0.7) nm; ^1^H and ^13^C NMR, see Table 1; HRESIMS *m/z* 422.1611 [M + H]^+^ (calcd for C_24_H_24_NO_6_, 422.1604).

### 4.5. Computational ECD Analysis of ***1***–***4***

Conformational analyses of **1**–**4** were initially carried out using CONFLEX^®^ software (Conflex Corp., Tokyo, Japan) in the Merck molecular force field (MMFF) to obtain populated conformers. Geometry optimization and TDDFT calculations were implemented using TURBOMOLE 7.2 (COSMOLogic GmbH, Leverkusen, Germany) with the combination of the 6-31G/B3LYP level and with the consideration of the first 50 excitations for ten major conformers from each isomer. Then, the Boltzmann-averaged spectra of each isomer were generated using a Gaussian band shape with 0.3 eV (**1**), 0.25 eV (**2**), 0.3 eV (**3**), and 0.25 eV (**4**) exponential half widths with UV correction (**1**–20 nm, **2**–23 nm, **3**–6 nm, **4**–19 nm). 

### 4.6. Cell Culture Method

GFP-LC3-transfected stable HEK293 cells were kindly donated by Professor Junsoo Park (Yonsei University, Republic of Korea), and MCF-7 and HEK293 cells were obtained from ATCC (Manassas, VA, USA). The cells were cultured in DMEM (Welgene, Gyeongsan-si, Republic of Korea) supplemented with 10% FBS (Gibco, Grand Island, NY, USA), 100 U/mL penicillin and 100 μg/mL streptomycin at 37 °C under 5% CO_2_ conditions. The cells were subcultured every 2 days and maintained at a confluency of 70 to 80%. 

### 4.7. Cell Viability Assay

The MTT method was applied to measure cell viability. Cells were seeded into 96-well plates (1 × 10^4^ cells per well) and incubated for 24 h. After cells attached to the bottom of the well plate, the cells were washed with PBS and treated with various concentrations of the tested compounds. After 24 h of treatment, 20 μL of MTT solution (2 mg/mL) was added to each well. After the MTT solution was incubated for 4 h at 37 °C, the solution was discarded by suction. A formazan crystal was dissolved in 100 μL of DMSO to measure the absorbance at 570 nm. The analysis was performed to calculate the cell viability compared with the control group and treatment group. 

### 4.8. Confocal Microscopy Imaging

Cells were seeded on sterilized glass coverslips in the well plate. Then, the cells were transfected with GFP-mRFP-LC3 (ptf-LC3) constructs, which were obtained from Yonsei University (WonJu, Republic of Korea) using Lipofectamine 2000 (Thermo Fisher Scientific, Waltham, MA, USA) according to the following product manuals. After transfection, the various concentrations of drug treatments were performed, and the cells were fixed with 3.7% paraformaldehyde for 10 min. After fixation, the slides were washed three times with PBS, stained with DAPI, and mounted on slide glass to observe the cells. The images of autophagic flux by treated compounds were captured by a Confocal Scope TCS8 (LEICA, Wetzlar, Germany). 

### 4.9. Protein Expression Analysis by Western Blot

The cells were seeded onto six-well plates and incubated for 24 h. After the isolated compounds were treated, the cells were collected and washed with PBS three times. Proteins were extracted from collected cells with 100 μL lysis buffer [120 mM NaCl, 40 mM Tris (pH 8), and 0.1% NP40 (Nonidet P-40)] and centrifuged at 10,000 rpm for 15 min. The supernatants were collected from the lysates, and protein concentrations were analyzed using a BCA protein assay kit (Bio-Rad, Hercules, CA, USA). Sample buffer was used to adjust to the same protein concentration and boiled for 5 min. The protein samples were loaded at 30 µg per well and electrophoresed on 6 to 15% gradient SDS-polyacrylamide gels. Proteins in the gels were transferred onto PVDF membranes and incubated with primary antibodies. The membranes were further incubated with secondary anti-mouse or anti-rabbit antibodies. Finally, the protein bands were detected using enhanced chemiluminescence Western blotting detection ECL buffer. Protein bands were quantified by ImageJ software.

## 5. Conclusions

In the present study, five dimeric benzylisoquinolines, three aporphines, and two benzylisoquinoline alkaloids were isolated from the stems of *L. scandens*, and their bioactivities were tested for the regulation of autophagy. Among the isolated compounds, **1**, **2**, and **4** showed good potencies for the inhibition of autophagic flux. These results suggest that *L. scandens* has novel autophagy-regulating compounds and is a good candidate for developing natural autophagy inhibitors for the prevention and treatment of various diseases. 

## Figures and Tables

**Table 1:** ^1^H and ^13^C NMR Spectroscopic Data for Compounds **1**–**4**.

Position	1 ^a^		2 ^a^		3 ^b^		4 ^c^	
	*δ*h (Multi, *J* in Hz)	*δ* c	*δ*h (Multi, *J* in Hz)	*δ* c	*δ*h (Multi, *J* in Hz)	*δ* c	*δ*h (Multi, *J* in Hz)	*δ* c
1	4.57, t (7.0)	57.4	4.57, t (7.2)	57.5	4.18, m	55.5	4.08, s	55.7
3	3.45, m	40.4	3.46, m	40.5	3.14, m 2.91, m	39.3	3.11, s 2.85, s	39.4
4	2.98, m	25.8	2.99, m	25.8	2.67, m	27.1	2.62, m	27.8
4a		123.6		123.6		124.2		124.7
5	6.69, s	112.6	6.75, s	112.6	6.61, s	112.1	6.59, s	112.1
6		149.3		149.3		146.4		146.2
7		146.6		146.6		144.4		144.3
8	6.47, s	114.4	6.46, s	114.4	6.53, s	113.5	6.55, s	113.5
8a		124.7		124.7		128.2		129.3
α	3.26, m 2.98, m	40.2	3.26, m 2.98, m	40.2	3.02, dd (13.8, 4.9) 2.83, dd (13.8, 8.4)	39.9	2.97, dd (13.3, 3.1) 2.78, dd (12.7, 8.9)	40.4
9		128.3		128.3		129.6		130.2
10	6.78, s	124.1	6.80, d (1.6)	124.1	6.97, d (1.9)	123.5	6.93, overlapped	123.5
11		144.3		144.3		140.7		141.1
12		150.1		150.1		147.8		147.7
13	6.96, d (8.2)	118.9	6.96, d (8.2)	118.9	6.95, overlapped	117.4	6.94, overlapped	117.2
14	7.03, d (7.4)	127.8	7.02, dd (8.2, 1.8)	127.8	7.03, dd (8.2, 1.6)	127.3	7.00, d (7.9)	126.9
1′		156.4		156.4		153.1		161.4
2′							7.87, br s	131.1
3′	8.27, d (6.3)	128.9	8.26, d (6.5)	128.8	8.32, d (5.5)	138.9	6.88, br s	115.3
4′	8.15, d (6.0)	124.1	8.15, d (6.4)	124.1	7.80, d (5.5)	121.1		125.3
4′a		137.9		137.9		132.6		
5′	7.63, s	107.5	7.63, s	107.5	7.28, s	105.6	6.88, br s	115.3
6′		158.3		158.3		152.9	7.87, br s	131.1
7′		152.4		152.4		149.1		167.4
8′	7.52, s	109.1	7.50, s	109.1	7.45, s	106.5		
8′a		122.1		122.1		121.9		
α′	6.60, s	71.5	6.61, s	71.4		193.5		
9′		135.3		135.5		130.2		
10′	7.33, d (8.4)	130.2	7.33, d (8.6)	130.2	7.77, d (8.8)	132.5		
11′	6.91, d (8.4)	118.3	6.91, d (8.6)	118.3	6.95, overlapped	115.5		
12′		160.1		160.1		162.5		
13′	6.91, d (8.4)	118.3	6.91, d (8.6)	118.3	6.95, overlapped	115.5		
14′	7.33, d (8.4)	130.2	7.33, d (8.6)	130.2	7.77, d (8.9)	132.5		
6-OCH_3_	3.79, s	56.4	3.84, s	56.4	3.68, s	55.5	3.71, s	55.5
6′-OCH_3_	4.11, s	57.4	4.12, s	57.4	3.96, s	55.8		

^a^ Measured in CD_3_OD at 500 MHz and 200 MHz. ^b^ Measured in DMSO-*d_6_* at 800 MHz and 200 MHz. ^c^ Measured DMSO-*d_6_* at 500 MHz and 125 MHz.

## Data Availability

Data is contained within the article and supplementary material.

## Supplementary Materials

The following supporting information can be downloaded at: https://www.mdpi.com/article/10.3390/ph15111332/s1, Figures S1–S26: 1D, 2D NMR, and HRESIMS spectra of compounds **1**–**4**. Figure S27: Experimental and calculated CD spectrum of compounds **3** and **4**. Figure S28: Cytotoxicity of compounds **1**–**10** on HEK293 cells. Figure S29: Morphological characteristics of *Limacia scandens* Lour. Figure S30: Uncropped western blot images.
