# Supplementary material for: Benzylisoquinoline Alkaloids from the Stems of Limacia scandens and Their Potential as Autophagy Inhibitors
Source: Pharmaceuticals (Basel). 2022 Oct 28;15(11):1332. doi: 10.3390/ph15111332 (PMC9693505; doi:10.3390/ph15111332)
Supplement: Supplementary file 1 [file pharmaceuticals-15-01332-s001.zip › pharmaceuticals-1955232-supplementary.pdf]

Supplementary Materials

Benzylisoquinoline Alkaloids from the Stems of *Limacia scandens* and Their Potential as Autophagy Inhibitors

Hee Ju Lee [1,2], Eun Jin Park [2], Byeol Ryu [2], Hyo Moon Cho [2], Duc-Trong Nghiem [3], Ha-Thanh-Tung Pham [4], Cheol-Ho Pan [1], and Won Keun Oh [2,*]

[1] Natural Product Informatics Research Center, Korea Institute of Science and Technology, Gangneung, 25451, Republic of Korea

[2] Korea Bioactive Natural Material Bank, Research Institute of Pharmaceutical Sciences, College of Pharmacy, Seoul National University, Seoul 08826, Republic of Korea

[3] Department of Botany, Hanoi University of Pharmacy, Hanoi 000084, Vietnam

[4] Faculty of Pharmacy, PHENIKAA University, Yen Nghia, Ha Dong, Hanoi 12116, Vietnam

[*] Correspondence: wkoh1@snu.ac.kr (W.K.O.)

Table of Contents

Figure S1. ^{1}H NMR spectrum (CD_3OD, 500 HMZ) of compound **1**.

Figure S2. ^{13}C NMR spectrum (CD_3OD, 200 HMZ) of compound **1**.

Figure S3. HSQC spectrum (CD_3OD, 500 HMZ) of compound **1**.

Figure S4. HMBC spectrum (CD_3OD, 500 HMZ) of compound **1**.

Figure S5. ^{1}H-^{1}H COSY spectrum (CD_3OD, 500 HMZ) of compound **1**.

Figure S6. ROESY spectrum (CD_3OD, 500 HMZ) of compound **1**.

Figure S7. HR-ESI-MS of compound **1**.

Figure S8. ^{1}H NMR spectrum (CD_3OD, 500 HMZ) of compound **2**.

Figure S9. ^{13}C NMR spectrum (CD_3OD, 200 HMZ) of compound **2**.

Figure S10. HSQC spectrum (CD_3OD, 500 HMZ) of compound **2**.

Figure S11. HMBC spectrum (CD_3OD, 500 HMZ) of compound **2**.

Figure S12. ^{1}H-^{1}H COSY spectrum (CD_3OD, 500 HMZ) of compound **2**.

Figure S13. ROESY spectrum (CD_3OD, 500 HMZ) of compound **2**.

Figure S14. HR-ESI-MS of compound **2**.

Figure S15. ^{1}H NMR spectrum (DMSO-d_6, 800 MHz) of compound **3**.

Figure S16. ^{13}C NMR spectrum (DMSO-d_6, 200 MHz) of compound **3**.

Figure S17. HSQC spectrum (DMSO-d_6, 800 MHz) of compound **3**.

Figure S18. HMBC spectrum (DMSO-d_6, 800 MHz) of compound **3**.

Figure S19. NOESY spectrum (DMSO-d_6, 500 MHz) of compound **3**.

Figure S20. HR-ESI-MS of compound **3**.

Figure S21. ^{1}H NMR spectrum (DMSO-d_6, 500 MHz) of compound **4**.

Figure S22. ^{13}C NMR spectrum (DMSO-d_6, 125 MHz) of compound **4**.

Figure S23. HSQC spectrum (DMSO-d_6, 800 MHz) of compound **4**.

Figure S24. HMBC spectrum (DMSO-d_6, 800 MHz) of compound **4**.

Figure S25. NOESY spectrum (DMSO-d_6, 800 MHz) of compound **4**.

Figure S26. HR-ESI-MS of compound **4**.

Figure S27. Experimental and calculated CD spectrum of compounds **3** and **4**.

Figure S28. Cytotoxicity of compounds **1**–**10** on HEK293 cells. The compounds were treated at 20 μM for 24 h, the cytotoxicity was calculated compared with control group.

Figure S29. Morphological characteristics of *Limacia scandens* Lour. (A) The whole plant, (B) Abaxial leaf blade, (C) Inflorescences.

Figure S30. Uncropped western blot images. (A) LC3B level, (B) p62 level, (C) β-actin level.

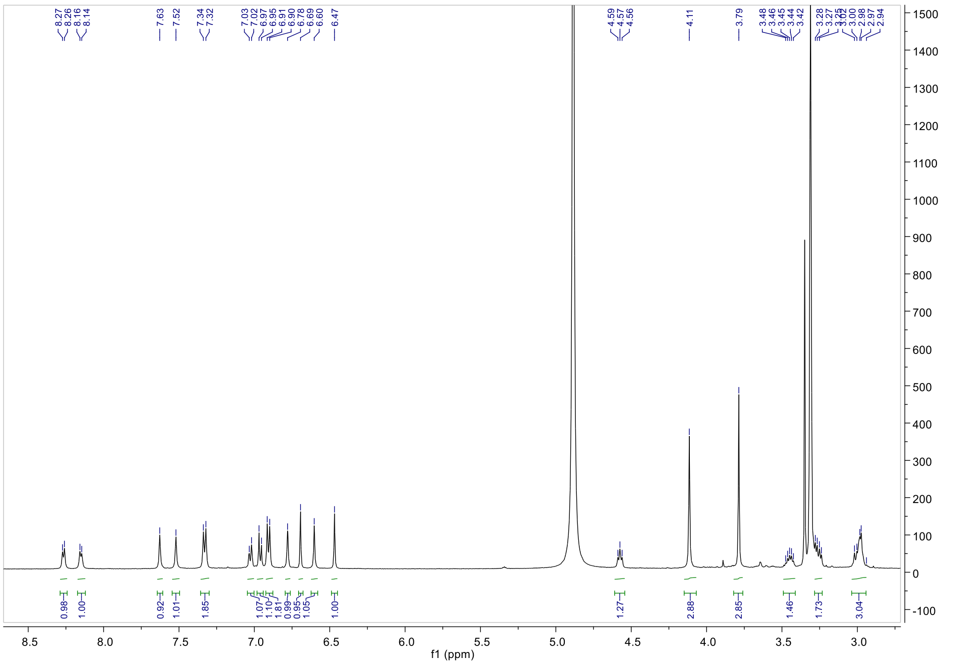

Figure S1. ^{1}H NMR spectrum (CD_3OD, 500 MHz) of compound **1**.

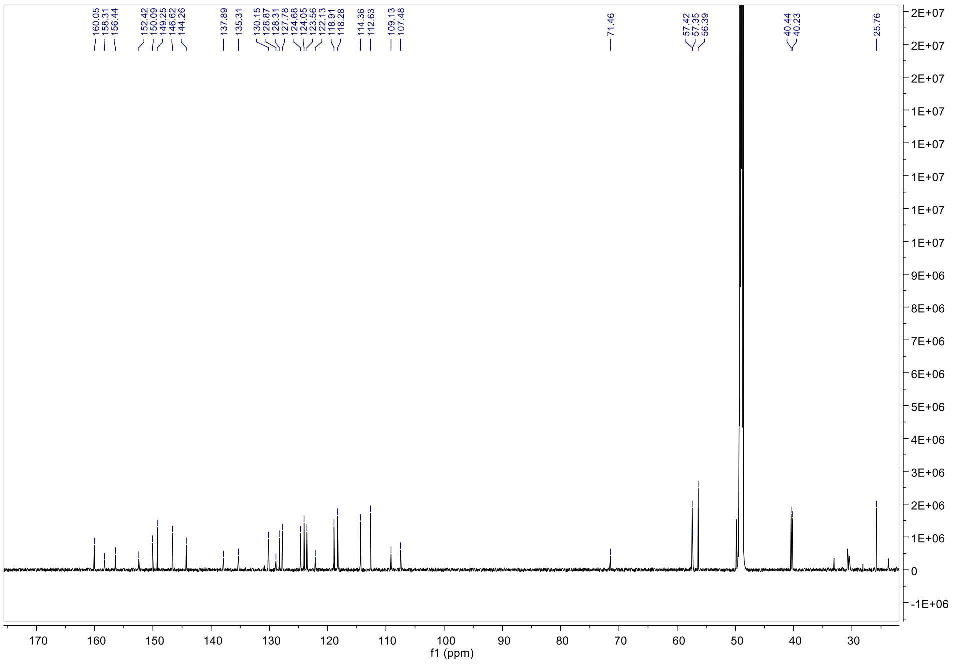

Figure S2. ^{13}C NMR spectrum (CD_3OD, 200 MHz) of compound **1**.

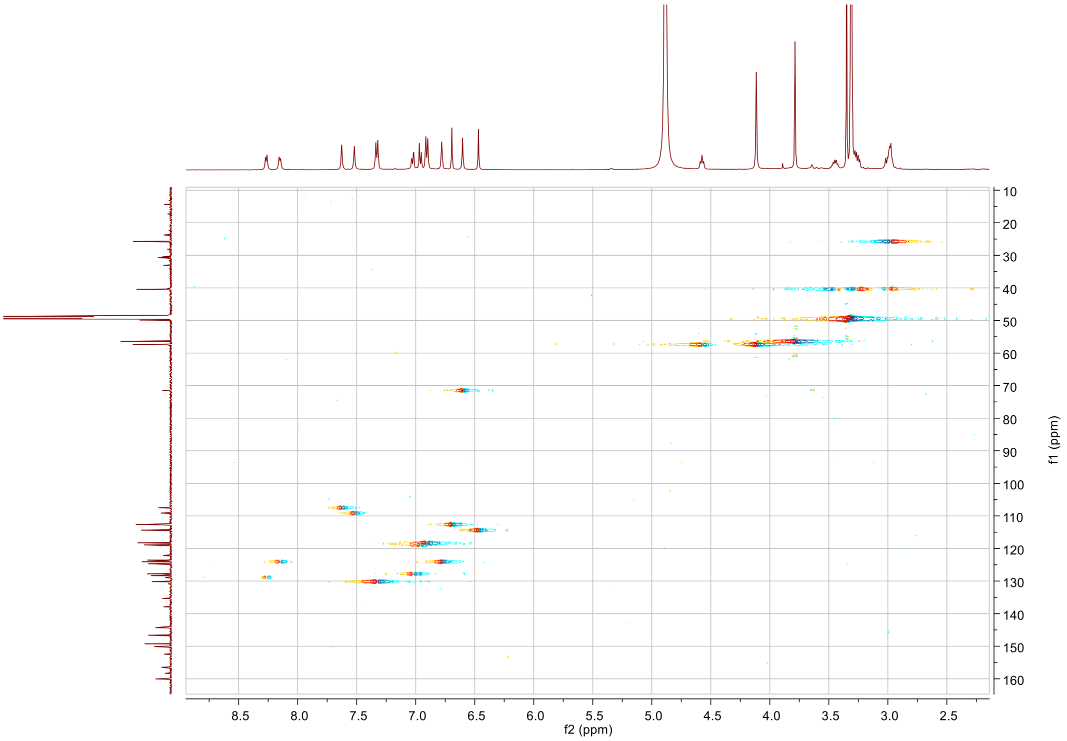

Figure S3. HSQC spectrum (CD_3OD, 500 MHz) of compound **1**.

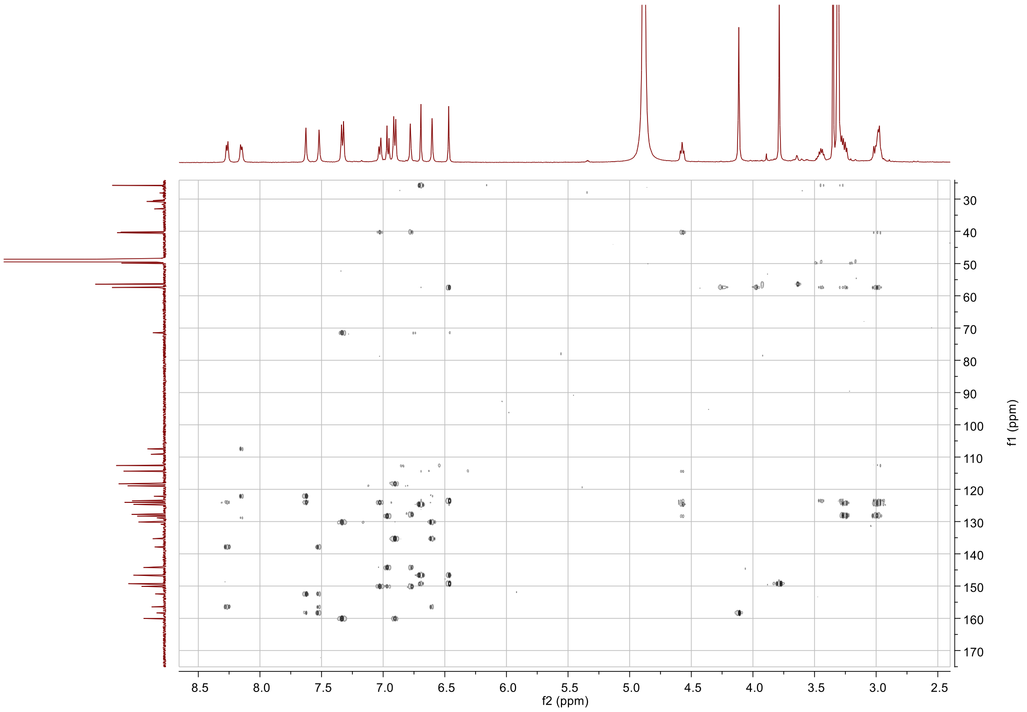

Figure S4. HMBC spectrum (CD_3OD, 500 MHz) of compound **1**.

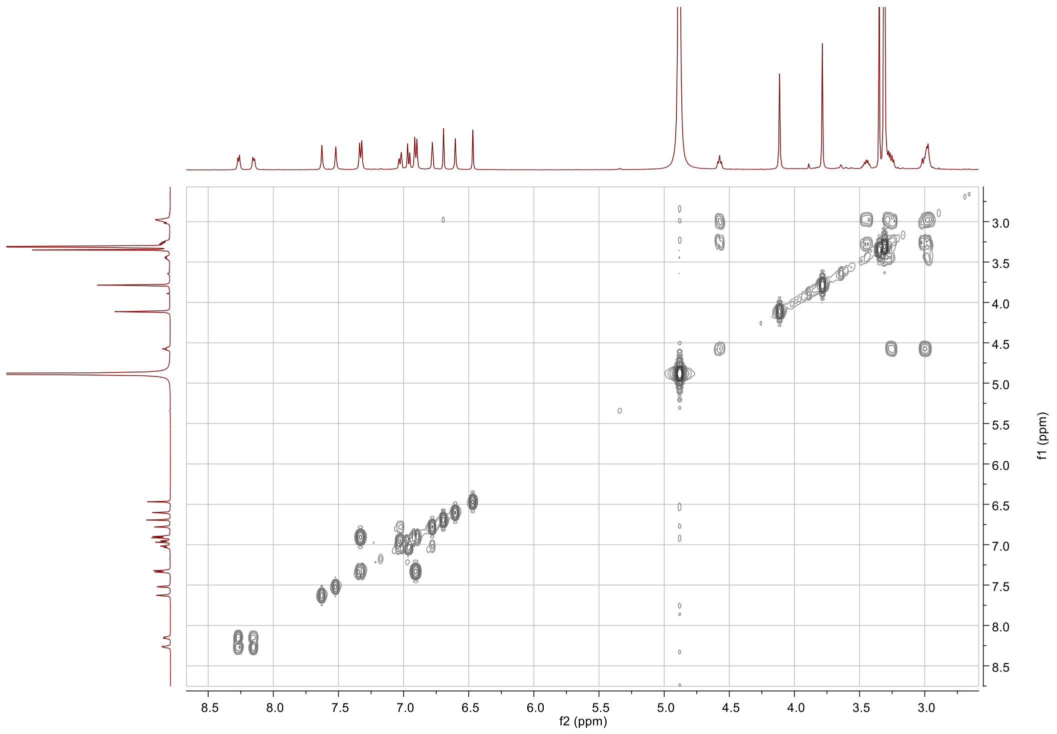

Figure S5. ^{1}H-^{1}H COSY spectrum (CD_3OD, 500 MHz) of compound **1**.

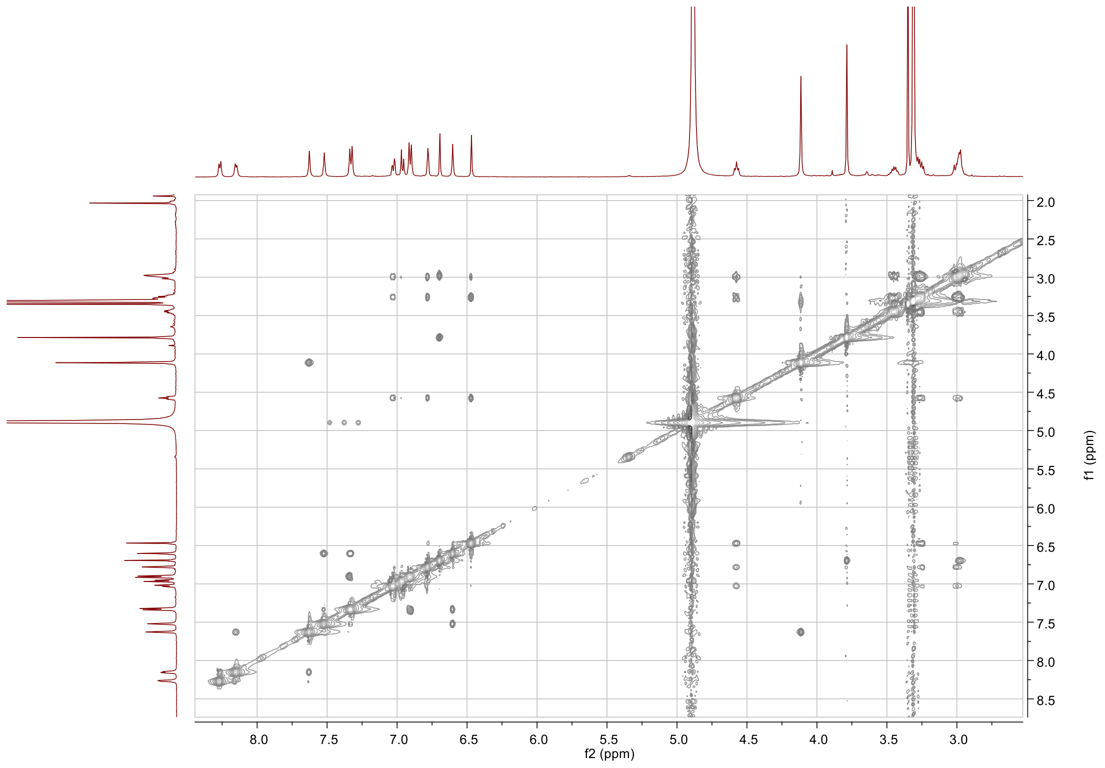

Figure S6. ROESY spectrum (CD_3OD, 500 MHz) of compound **1**.

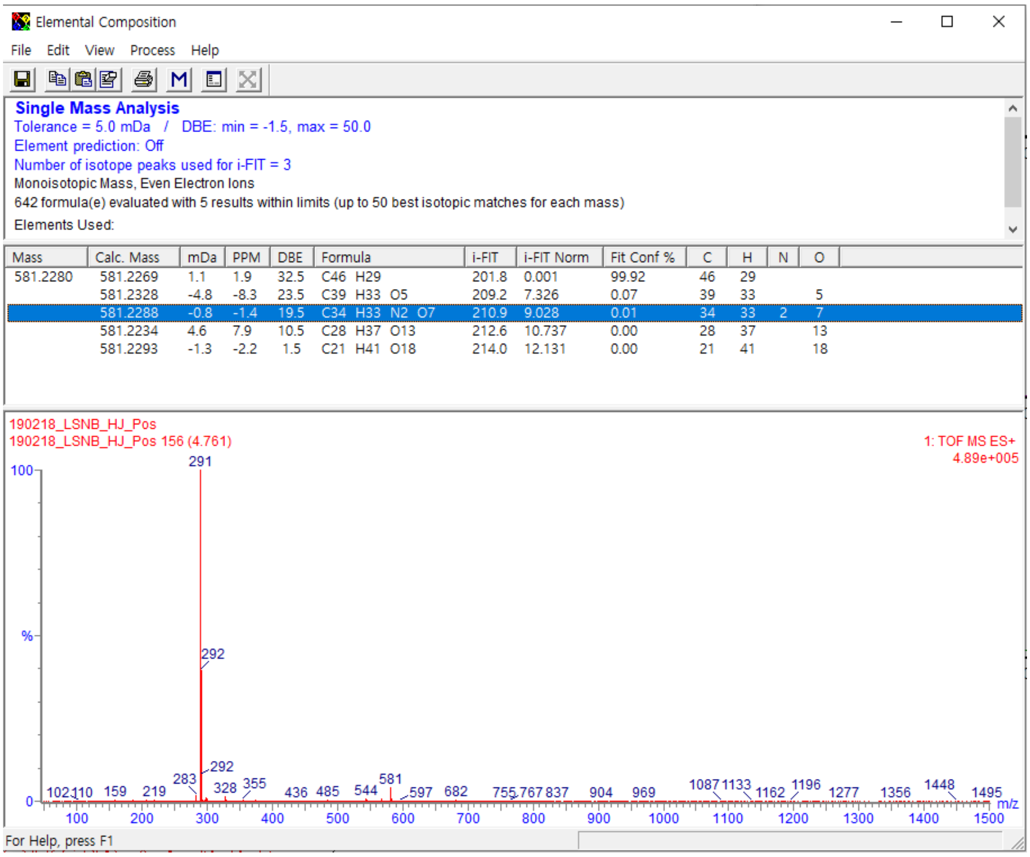

Figure S7. HR-ESI-MS of compound **1**.

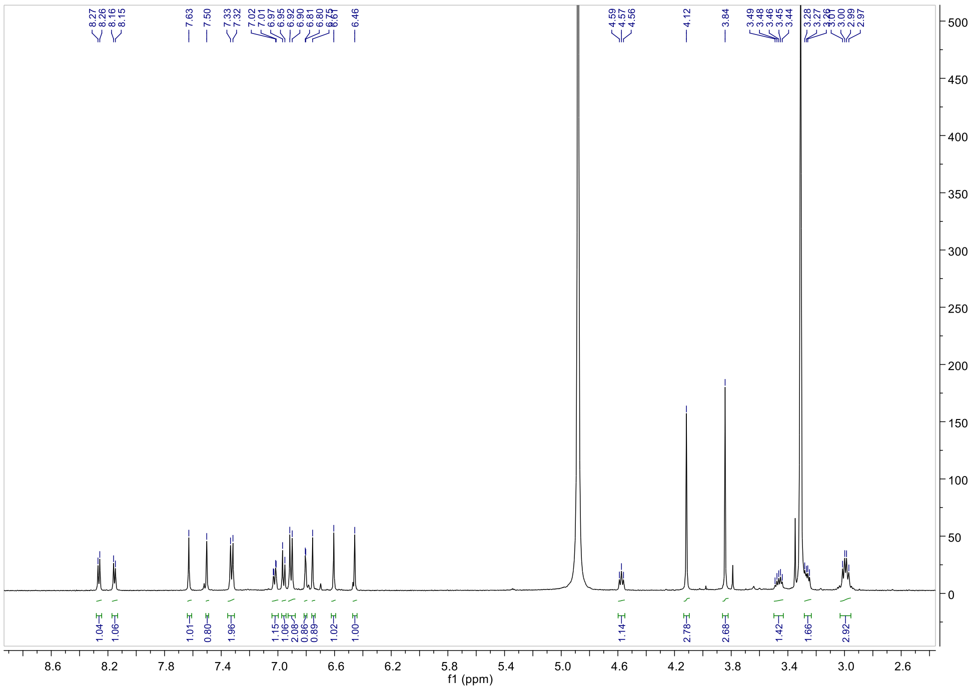

Figure S8. ^{1}H NMR spectrum (CD_3OD, 500 MHz) of compound **2**.

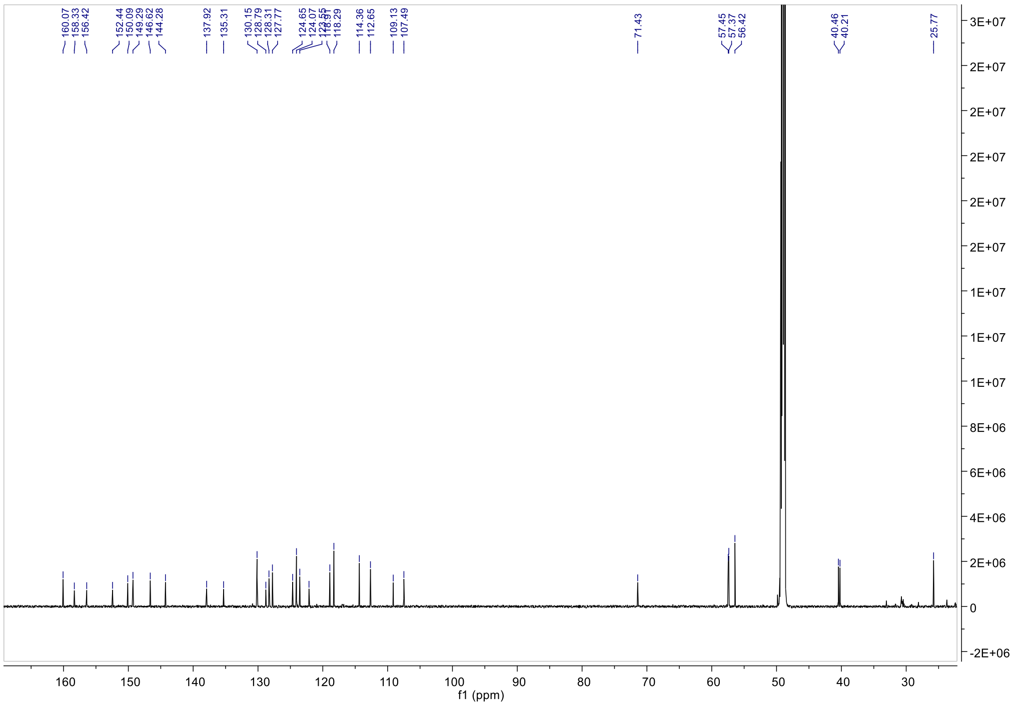

Figure S9. ^{13}C NMR spectrum (CD_3OD, 200 MHz) of compound **2**.

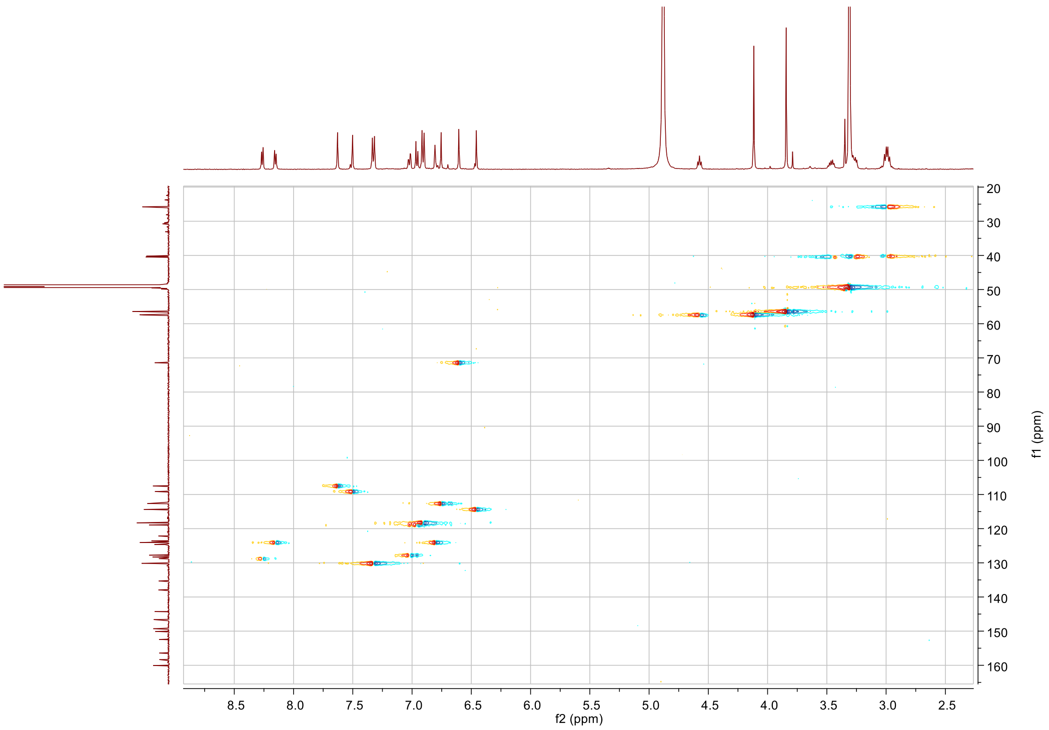

Figure S10. HSQC spectrum (CD_3OD, 500 MHz) of compound **2**.

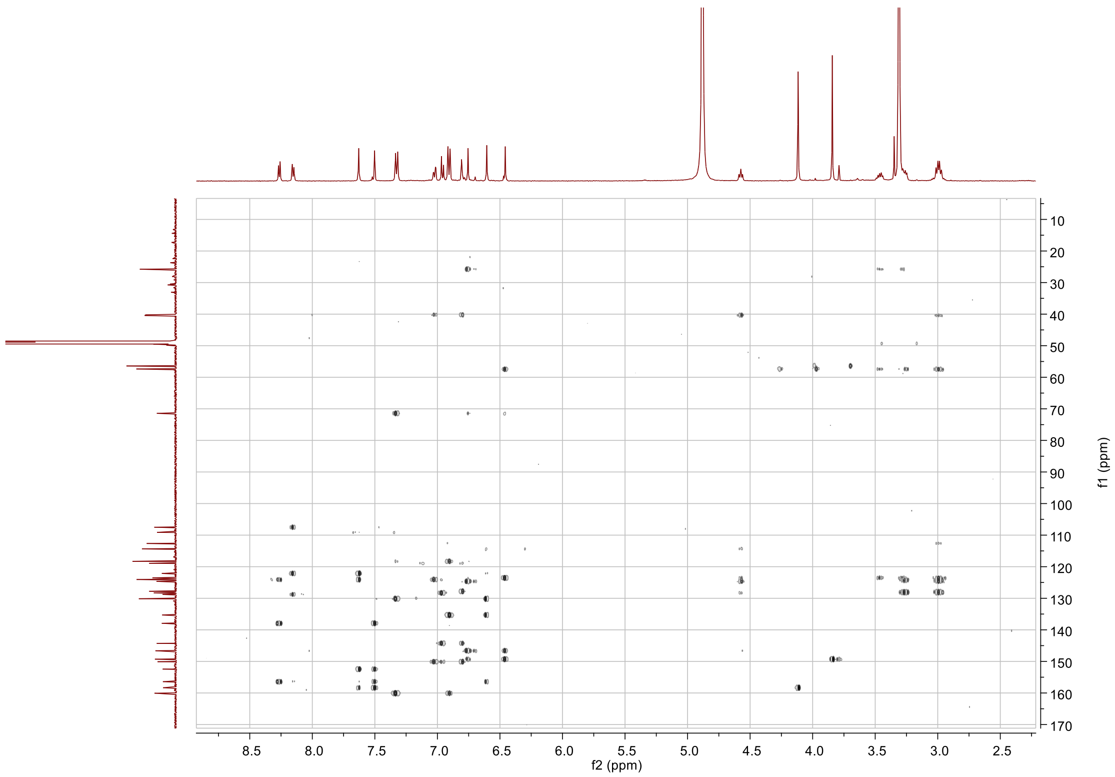

Figure S11. HMBC spectrum (CD_3OD, 500 MHz) of compound **2**.

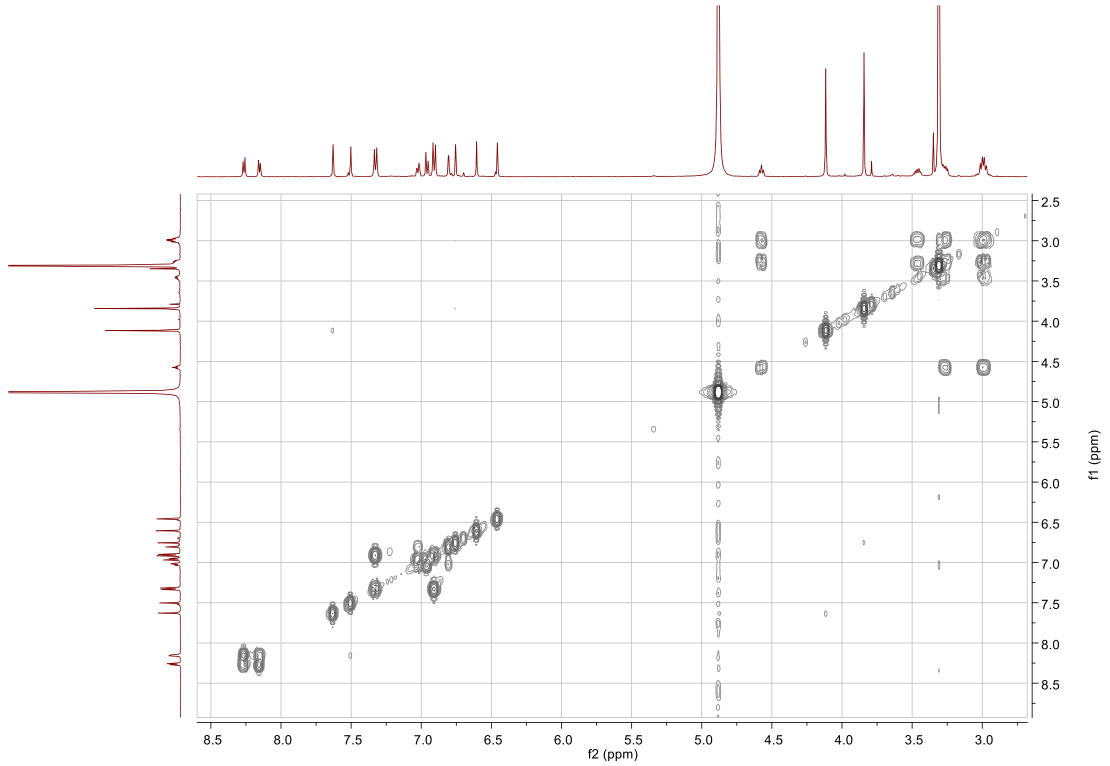

Figure S12. ^1H-^1H COSY spectrum (CD_3OD, 500 MHz) of compound **2**.

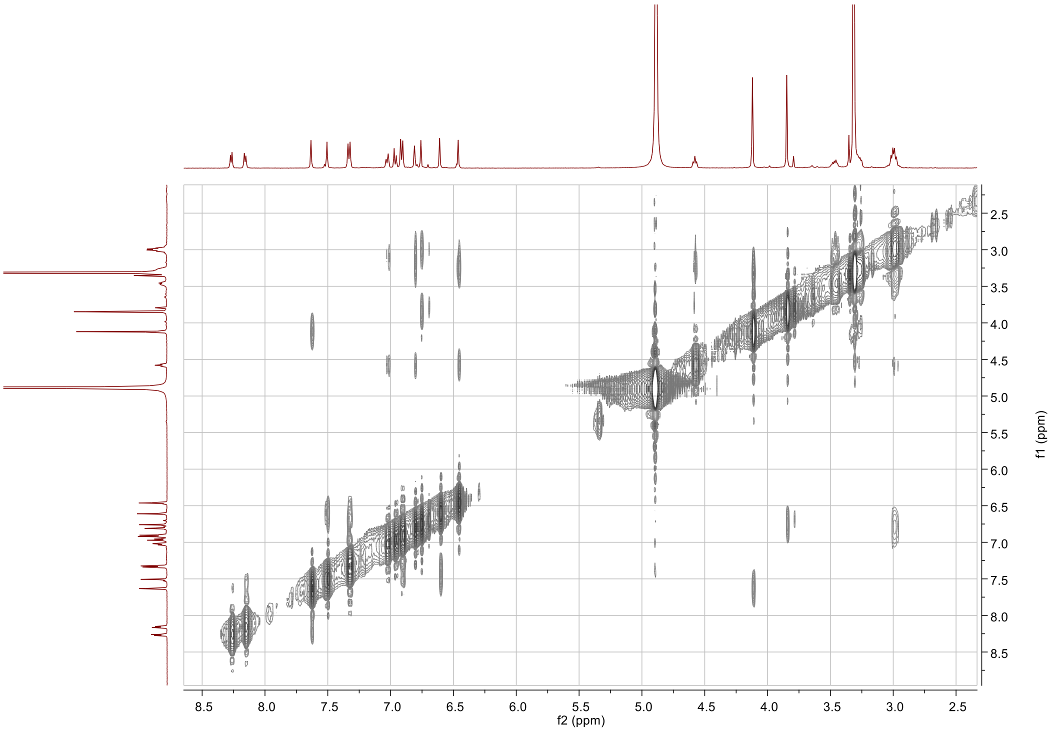

Figure S13. ROESY spectrum (CD3OD, 500 MHz) of compound **2**.

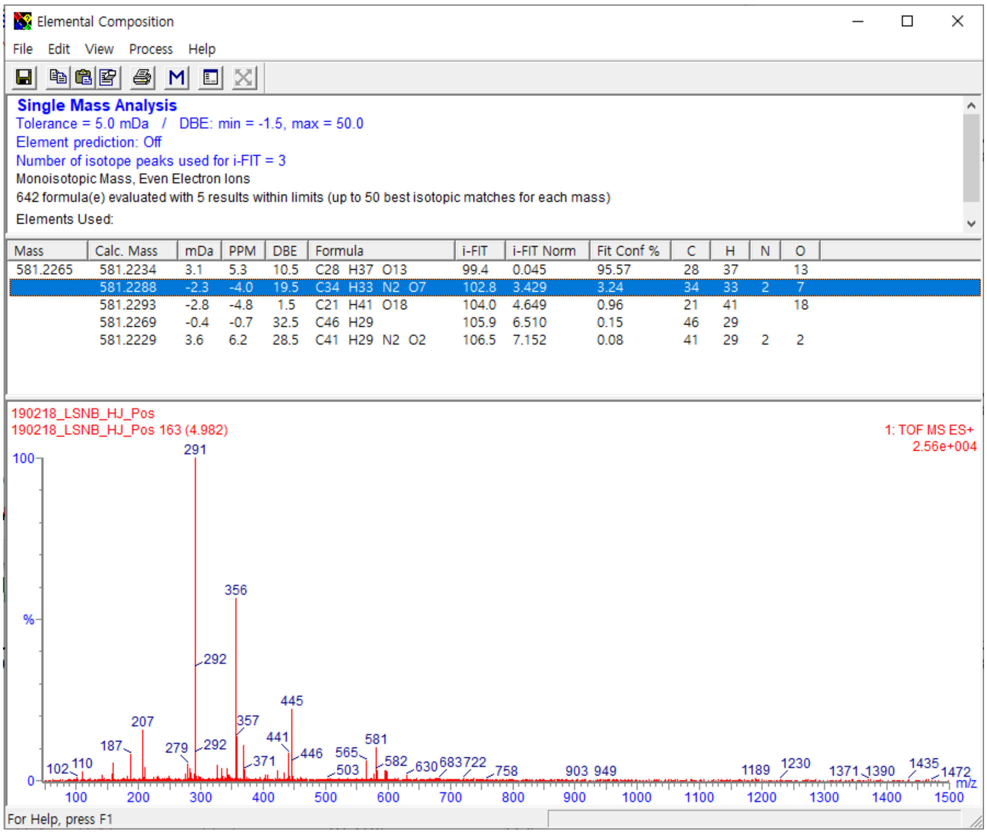

Mass	Calc. Mass	mDa	PPM	DBE	Formula	i-FIT	i-FIT Norm	Fit Conf %	C	H	N	O
581.2265	581.2234	3.1	5.3	10.5	C28 H37 O13	99.4	0.045	95.57	28	37		13
	581.2288	-2.3	-4.0	19.5	C34 H33 N2 O7	102.8	3.429	3.24	34	33	2	7
	581.2293	-2.8	-4.8	1.5	C21 H41 O18	104.0	4.649	0.96	21	41		18
	581.2269	-0.4	-0.7	32.5	C46 H29	105.9	6.510	0.15	46	29		
	581.2229	3.6	6.2	28.5	C41 H29 N2 O2	106.5	7.152	0.08	41	29	2	2

Figure S14. HR-ESI-MS of compound **2**.

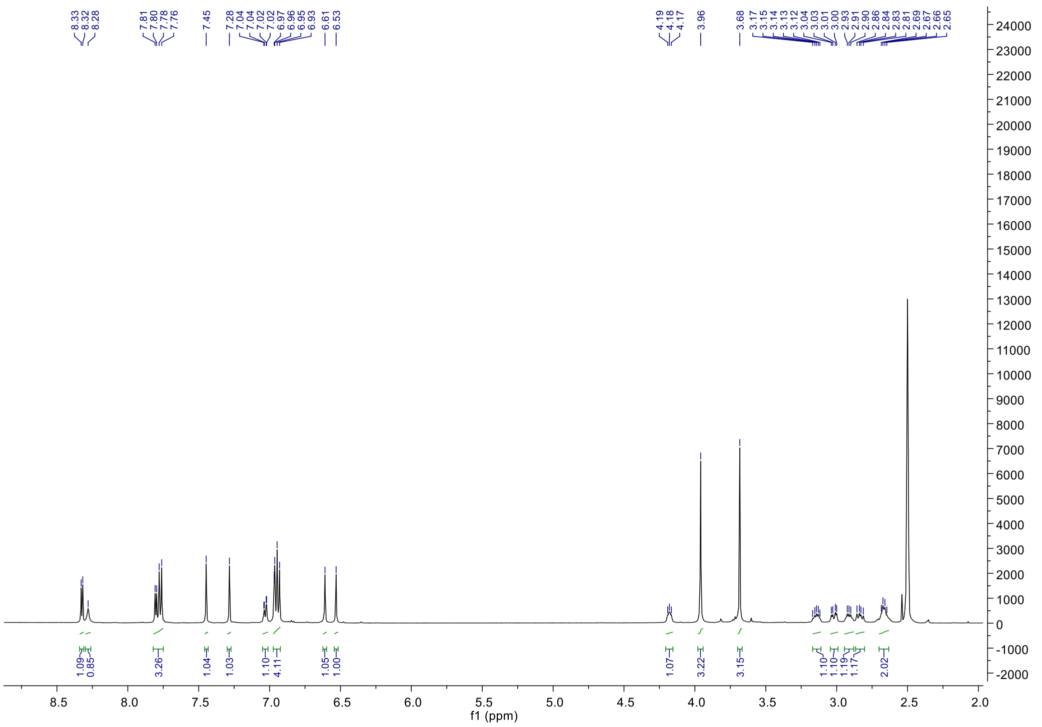

Figure S15. ^{1}H NMR spectrum (DMSO-d_6, 800 MHz) of compound **3**.

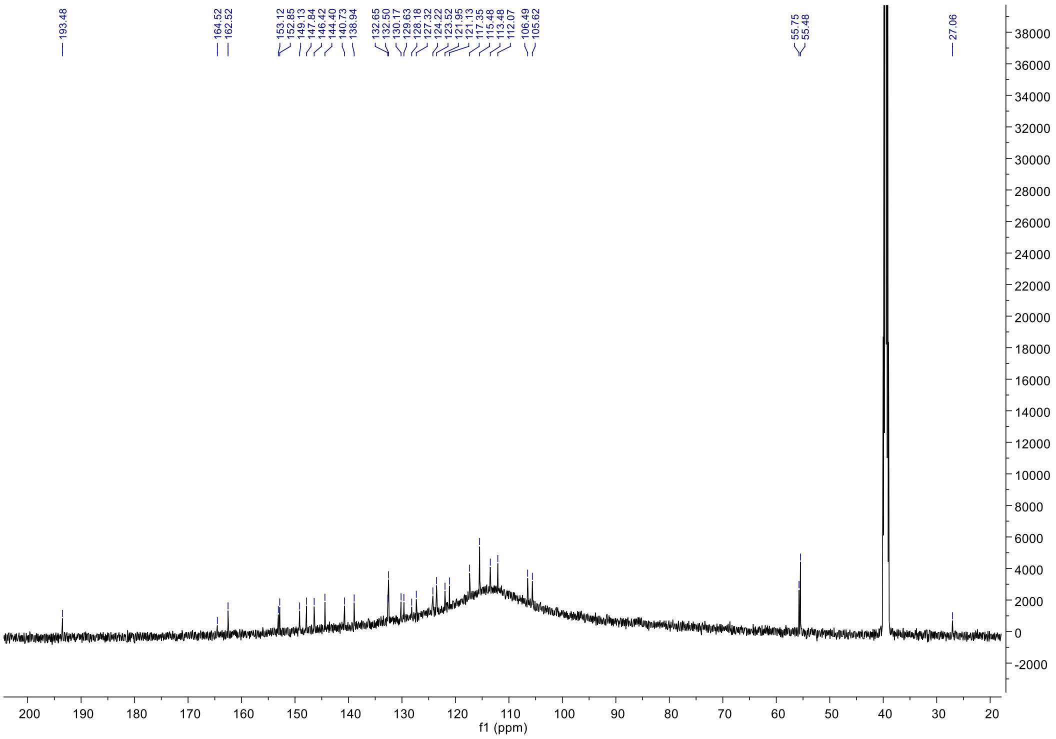

Figure S16. ^{13}C NMR spectrum (DMSO-d_6, 200 MHz) of compound **3**.

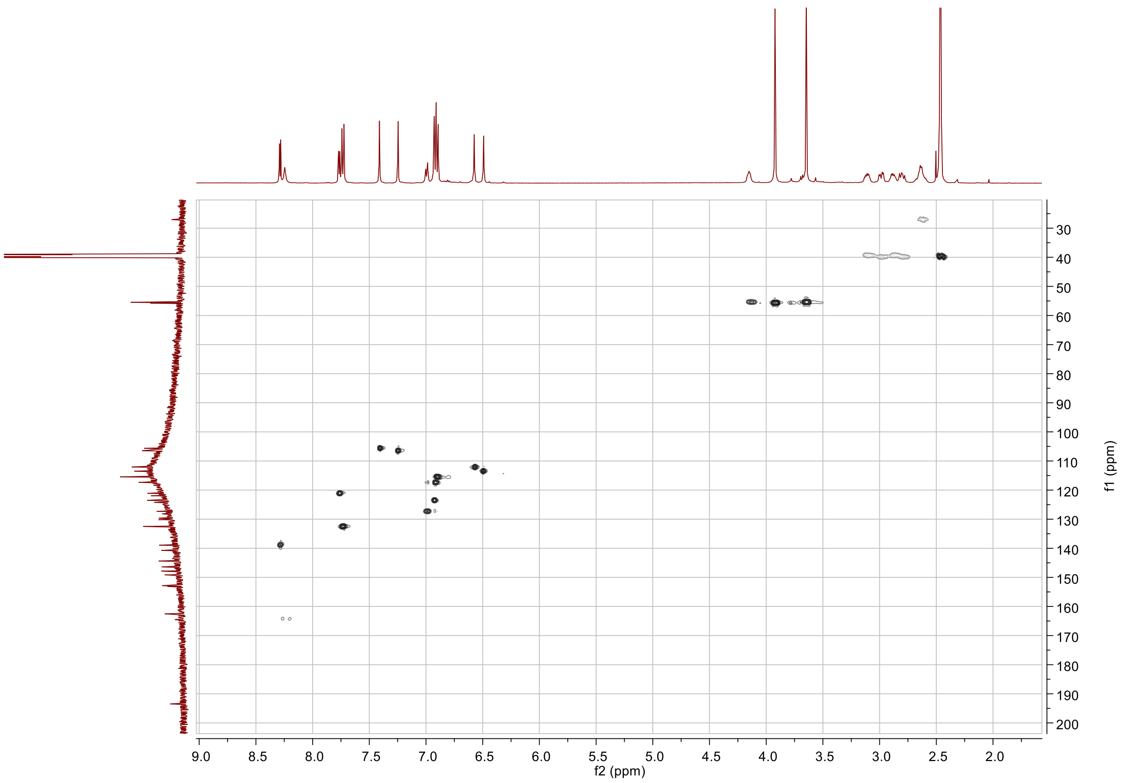

Figure S17. HSQC spectrum (DMSO-d_6, 800 MHz) of compound **3**.

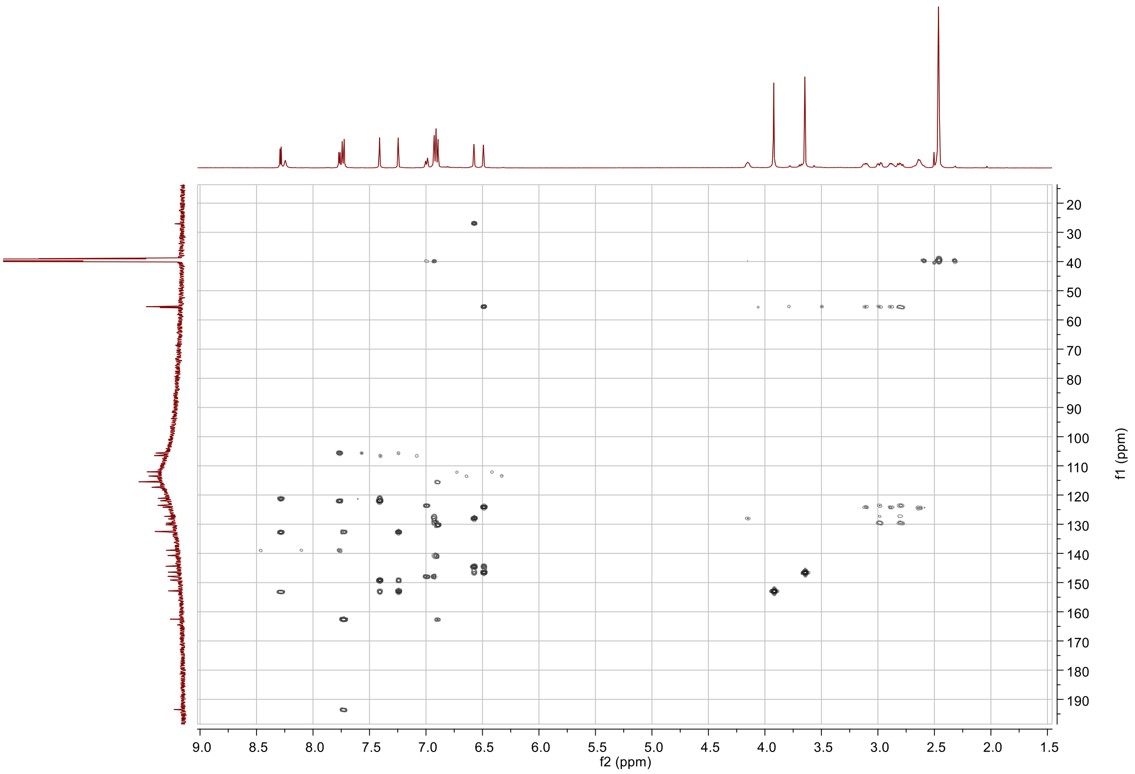

Figure S18. HMBC spectrum (DMSO-d_6, 800 MHz) of compound **3**.

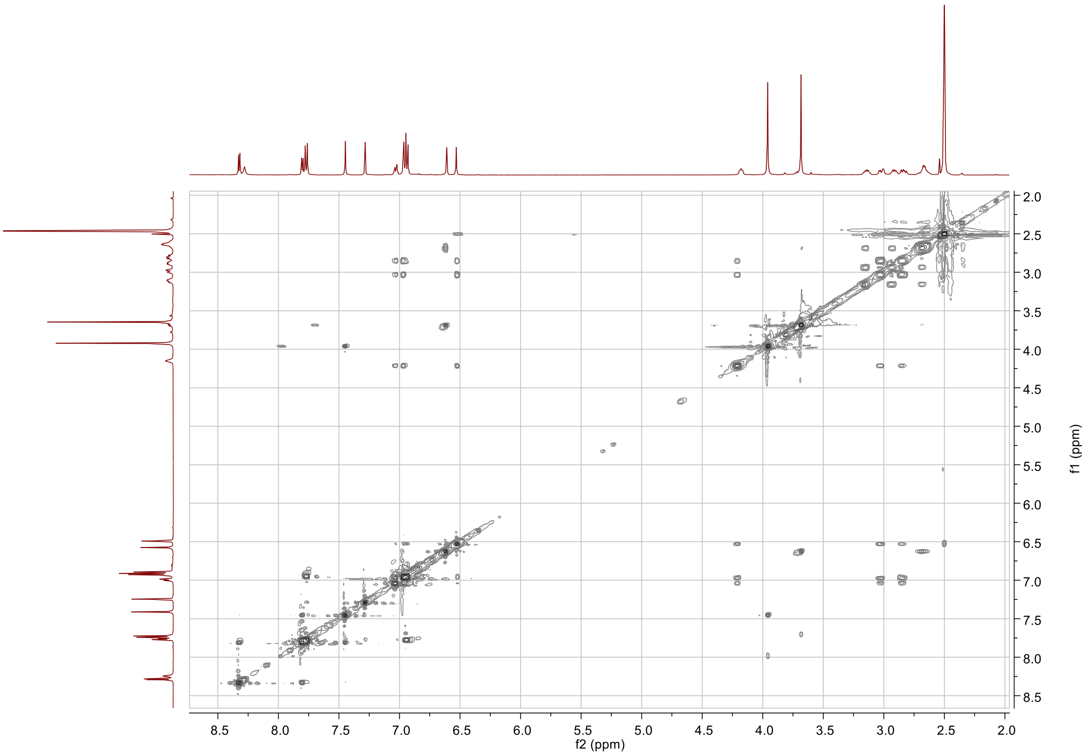

Figure S19. NOESY spectrum (DMSO-d_6, 500 MHz) of compound **3**.

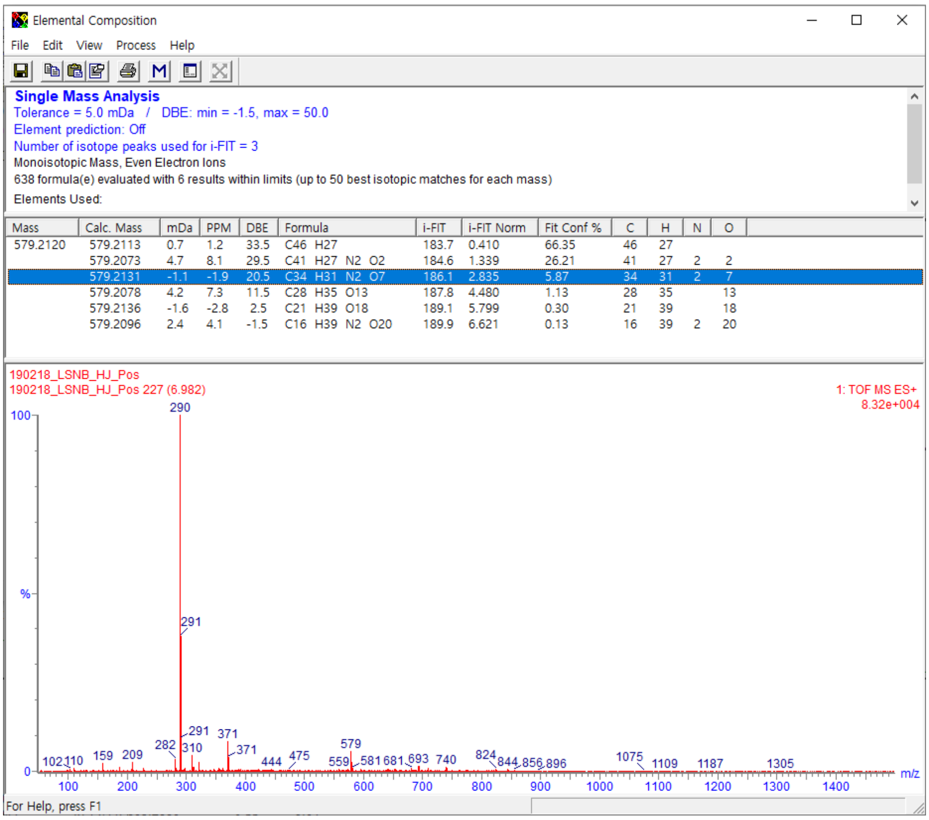

Mass	Calc. Mass	mDa	PPM	DBE	Formula	i-FIT	i-FIT Norm	Fit Conf %	C	H	N	O
579.2120	579.2113	0.7	1.2	33.5	C46 H27	183.7	0.410	66.35	46	27		
	579.2073	4.7	8.1	29.5	C41 H27 N2 O2	184.6	1.339	26.21	41	27	2	2
	579.2131	-1.1	-1.9	20.5	C34 H31 N2 O7	186.1	2.835	5.87	34	31	2	7
	579.2078	4.2	7.3	11.5	C28 H35 O13	187.8	4.480	1.13	28	35		13
	579.2136	-1.6	-2.8	2.5	C21 H39 O18	189.1	5.799	0.30	21	39		18
	579.2096	2.4	4.1	-1.5	C16 H39 N2 O20	189.9	6.621	0.13	16	39	2	20

Figure S20. HR-ESI-MS of compound **3**.

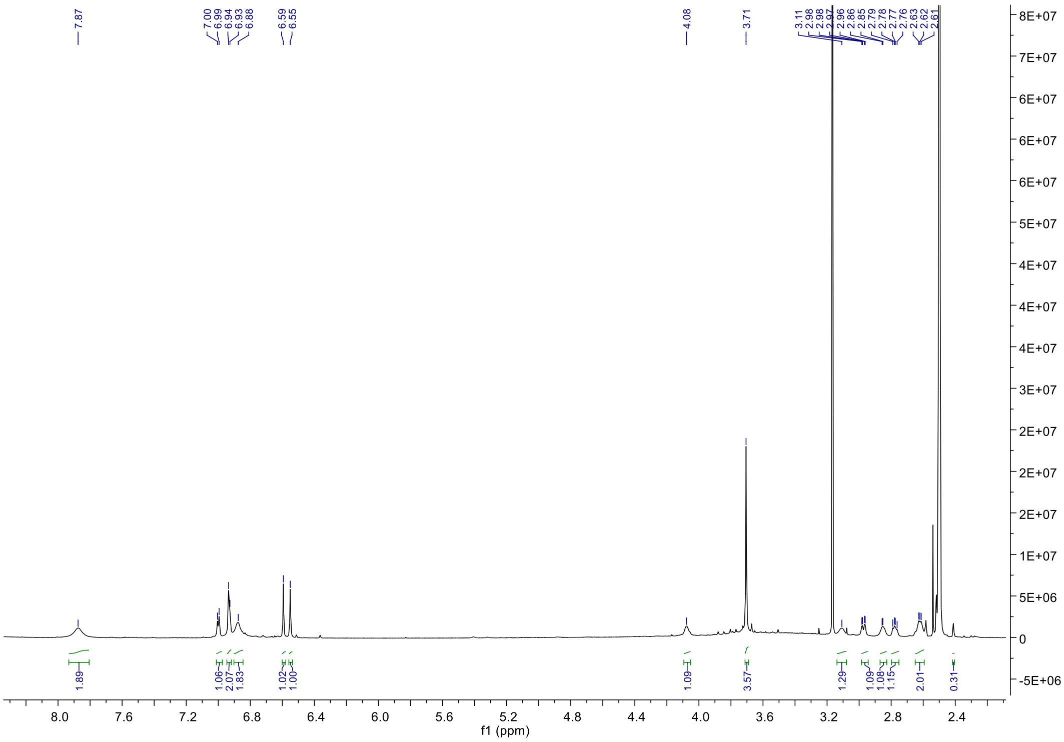

Figure S21. ^{1}H NMR spectrum (DMSO-d_6, 500 MHz) of compound **4**.

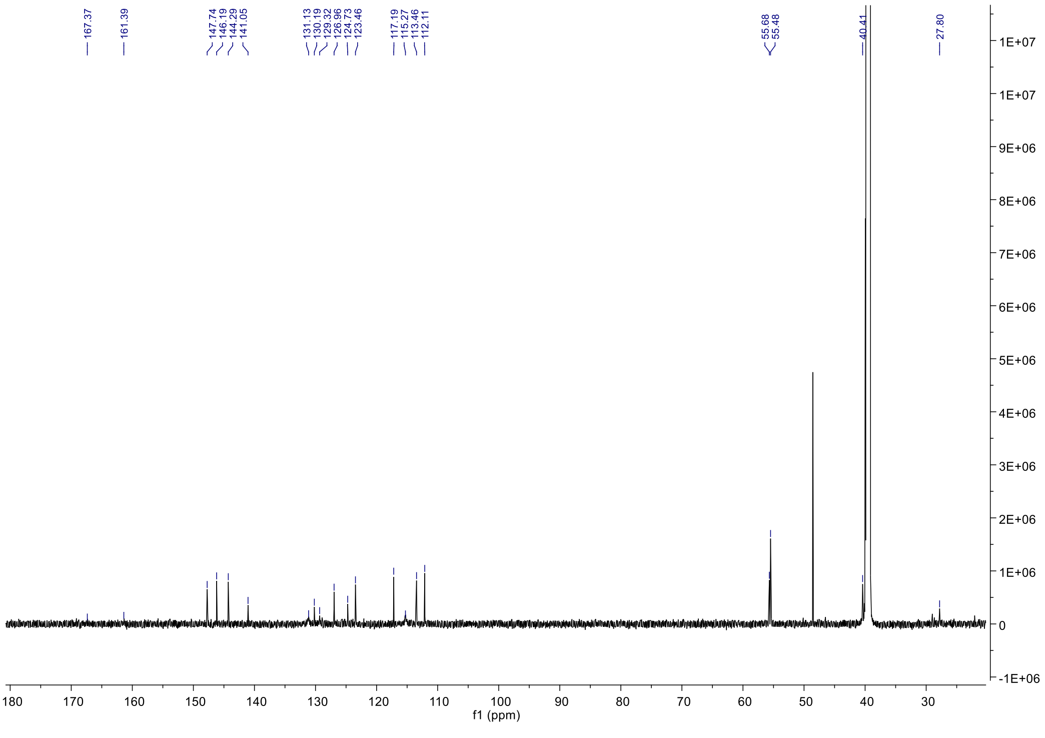

Figure S22. ^{13}C NMR spectrum (DMSO-d_6, 125 MHz) of compound **4**.

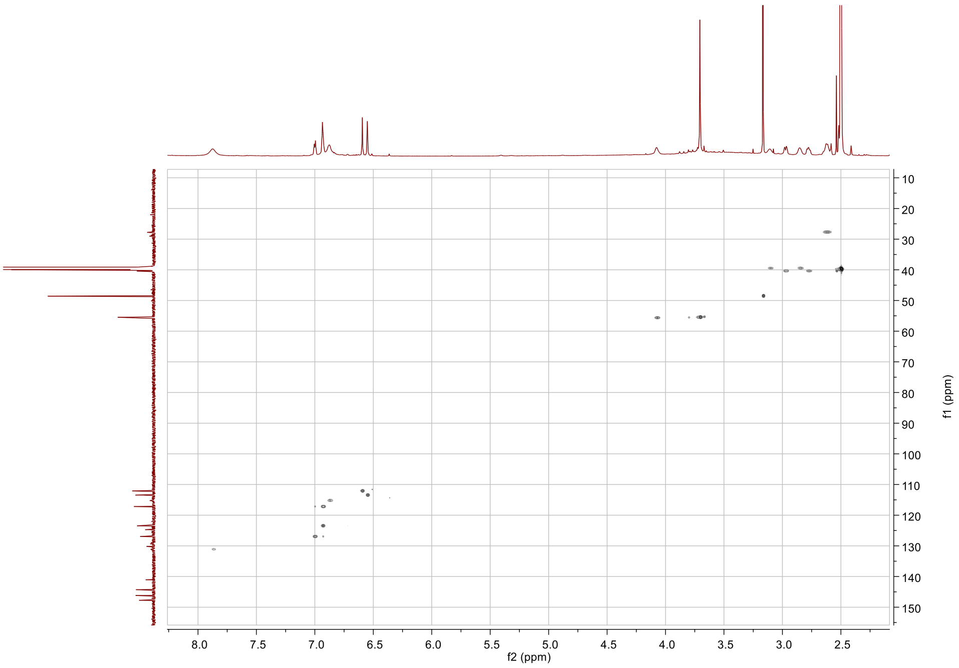

Figure S23. HSQC spectrum (DMSO-d_6, 800 MHz) of compound **4**.

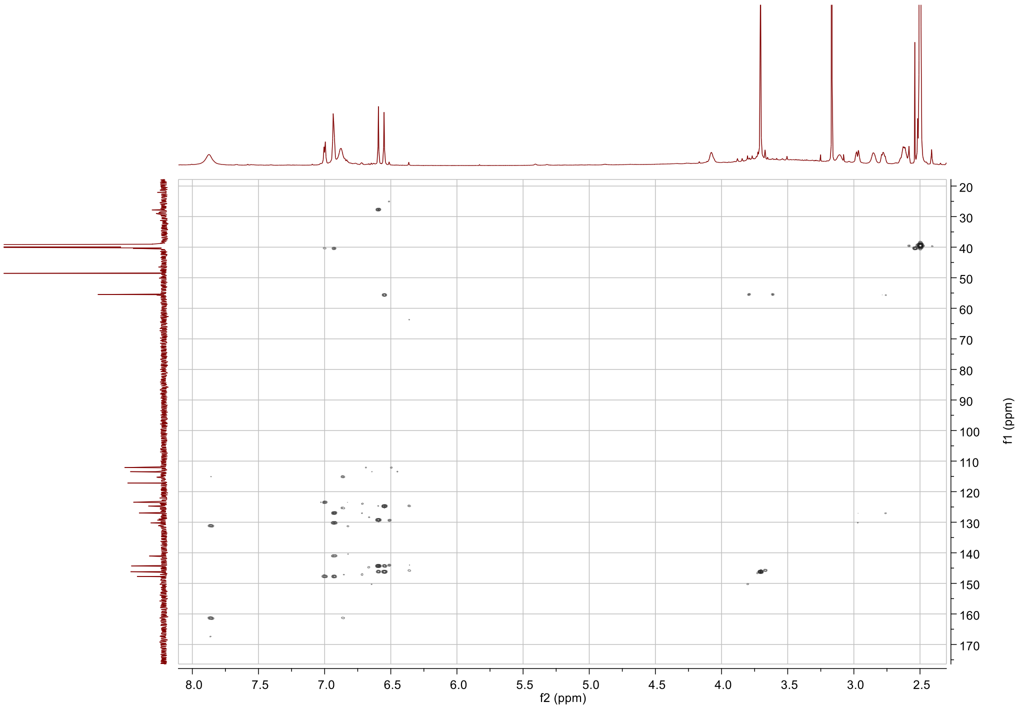

Figure S24. HMBC spectrum (DMSO-d_6, 800 MHz) of compound **4**.

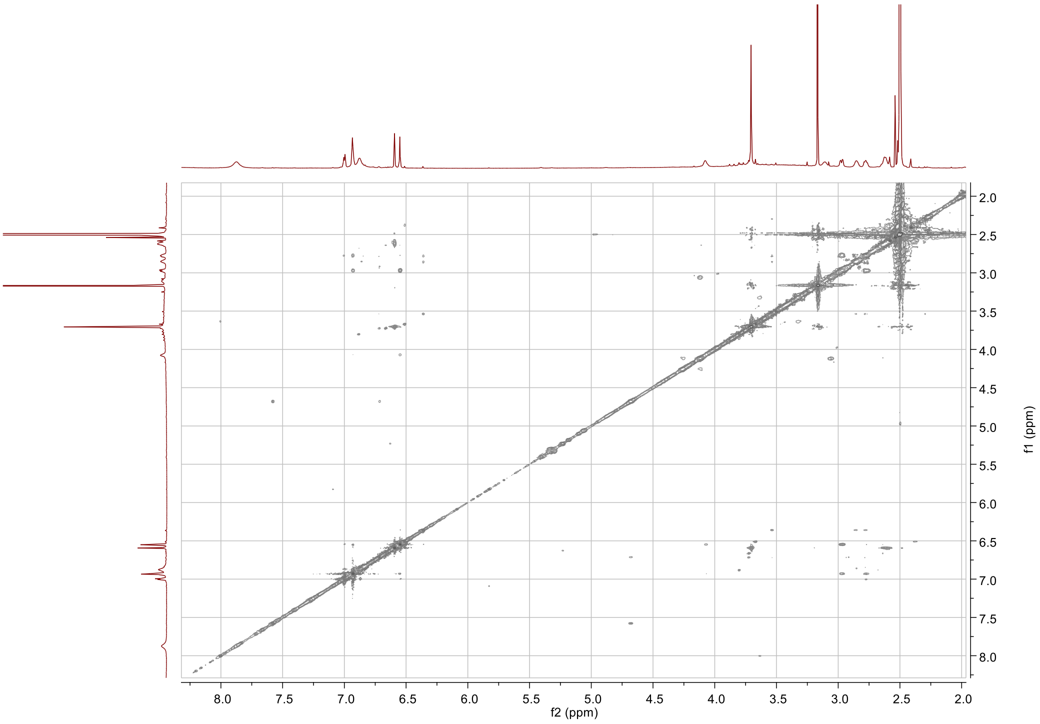

Figure S25. ROESY spectrum (DMSO-d_6, 800 MHz) of compound **4**.

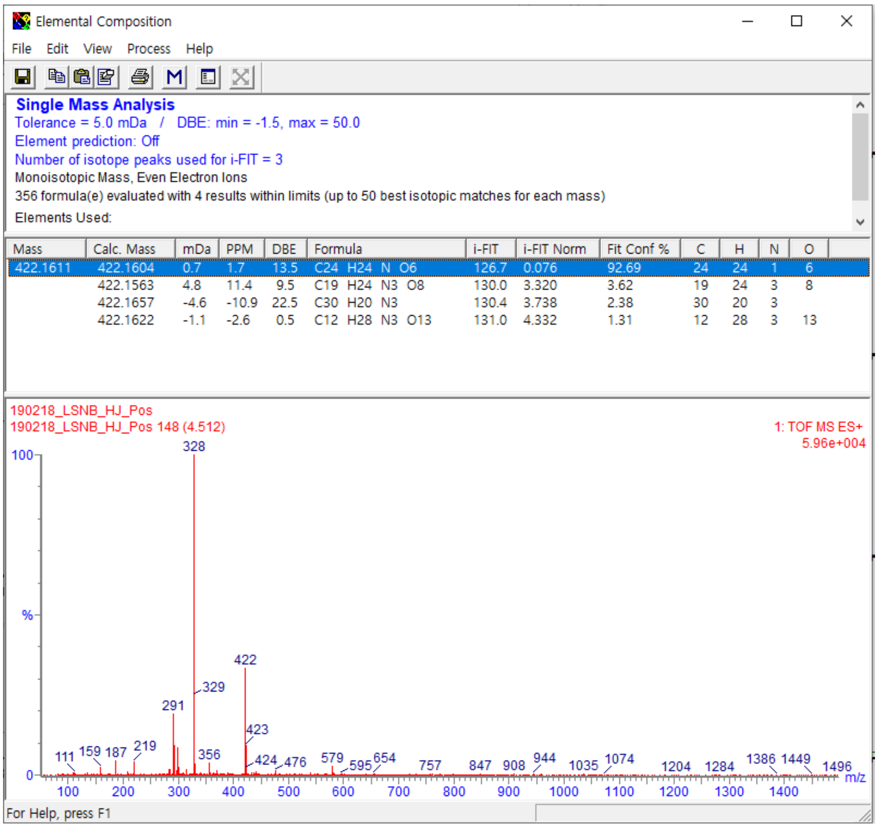

Mass	Calc. Mass	mDa	PPM	DBE	Formula	i-FIT	i-FIT Norm	Fit Conf %	C	H	N	O
422.1611	422.1604	0.7	1.7	13.5	C24 H24 N O6	126.7	0.076	92.69	24	24	1	6
	422.1563	4.8	11.4	9.5	C19 H24 N3 O8	130.0	3.320	3.62	19	24	3	8
	422.1657	-4.6	-10.9	22.5	C30 H20 N3	130.4	3.738	2.38	30	20	3	
	422.1622	-1.1	-2.6	0.5	C12 H28 N3 O13	131.0	4.332	1.31	12	28	3	13

Figure S26. HR-ESI-MS of compound **4**.

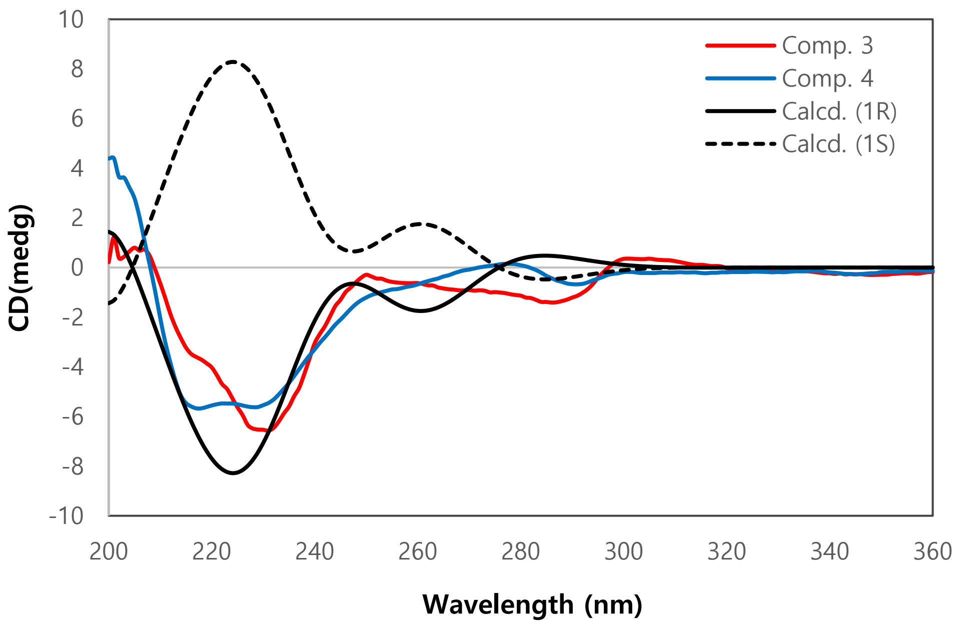

Figure S27. Experimental and calculated CD spectrum of compounds **3** and **4**.

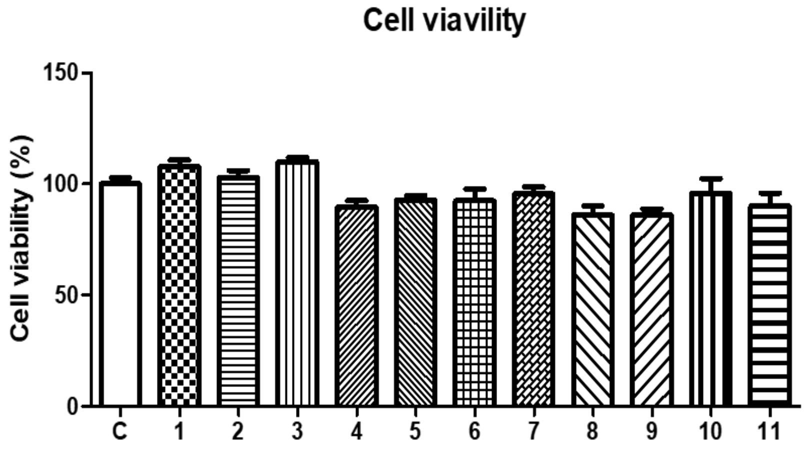

Figure S28. Cytotoxicity of compounds **1**–**10** on HEK293 cells. The compounds were treated at 20 μM for 24 h, the cytotoxicity was calculated compared with control group.

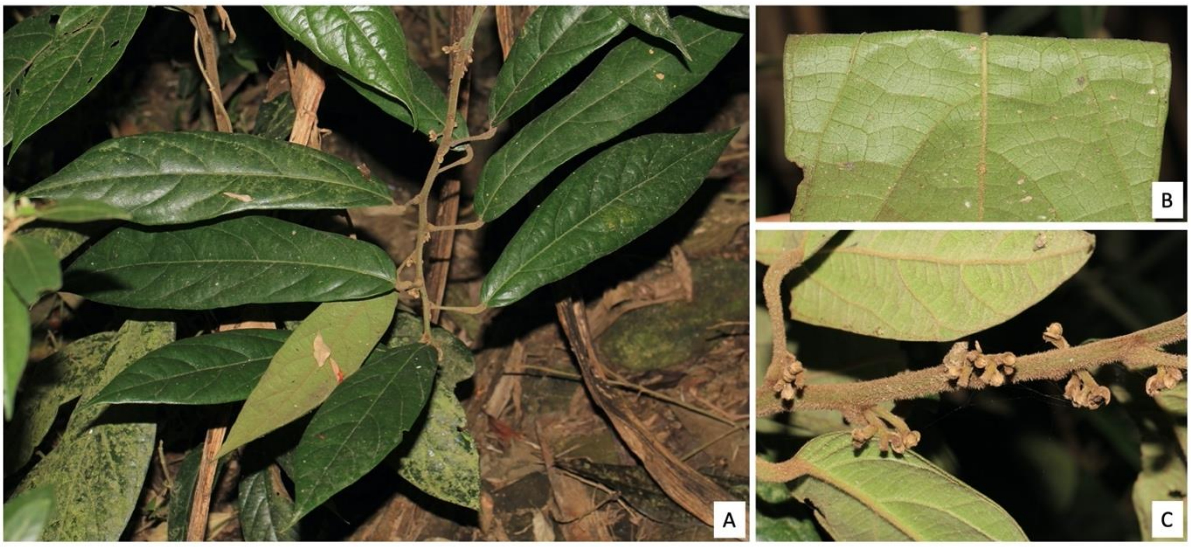

Figure S29. Morphological characteristics of Limacia scandens Lour. (A) The whole plant, (B) Abaxial leaf blade, (C) Inflorescences.

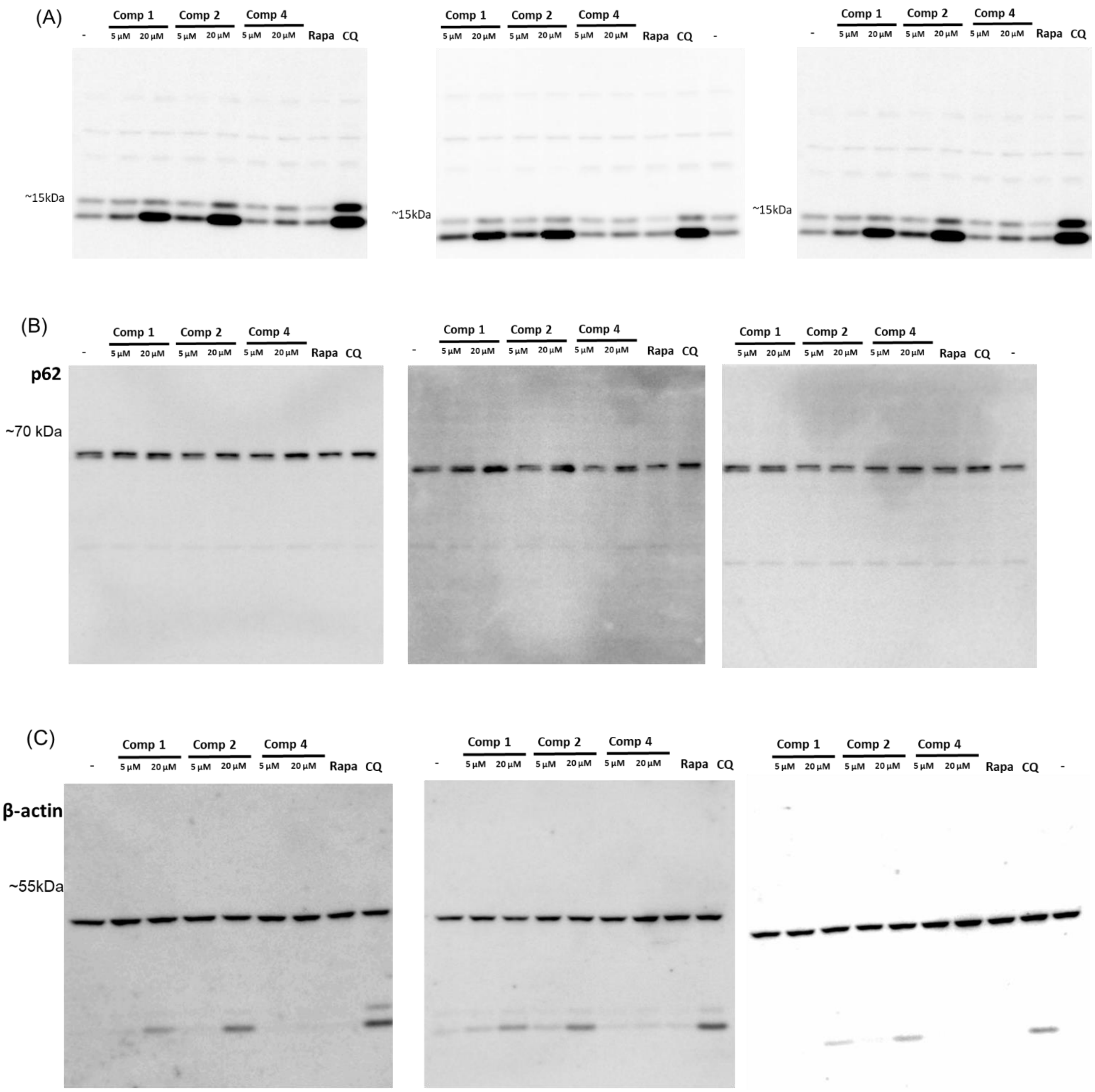

Figure S30. Uncropped western blot images. (A) LC3B level, (B) p62 level, (C) β-actin level.
